# Rearrangement and evolution of mitochondrial genomes in Thysanoptera (Insecta)

**DOI:** 10.1038/s41598-020-57705-4

**Published:** 2020-01-20

**Authors:** Kaomud Tyagi, Rajasree Chakraborty, Stephen L. Cameron, Andrew D. Sweet, Kailash Chandra, Vikas Kumar

**Affiliations:** 1 Centre for DNA Taxonomy, Molecular Systematics Division, Zoological Survey of India, Kolkata, 750053 India; 2 Department of Entomology, Purdue University, West Lafayette, IN 47907 USA

**Keywords:** Phylogenetics, Next-generation sequencing

## Abstract

Prior to this study, complete mitochondrial genomes from Order Thysanoptera were restricted to a single family, the Thripidae, resulting in a biased view of their evolution. Here we present the sequences for the mitochondrial genomes of four additional thrips species, adding three extra families and an additional subfamily, thus greatly improving taxonomic coverage. Thrips mitochondrial genomes are marked by high rates of gene rearrangement, duplications of the control region and tRNA mutations. Derived features of mitochondrial tRNAs in thrips include gene duplications, anticodon mutations, loss of secondary structures and high gene translocation rates. Duplicated control regions are found in the Aeolothripidae and the ‘core’ Thripinae clade but do not appear to promote gene rearrangement as previously proposed. Phylogenetic analysis of thrips mitochondrial sequence data supports the monophyly of two suborders, a sister-group relationship between Stenurothripidae and Thripidae, and suggests a novel set of relationships between thripid genera. Ancestral state reconstructions indicate that genome rearrangements are common, with just eight gene blocks conserved between any thrips species and the ancestral insect mitochondrial genome. Conversely, 71 derived rearrangements are shared between at least two species, and 24 of these are unambiguous synapomorphies for clades identified by phylogenetic analysis. While the reconstructed sequence of genome rearrangements among the protein-coding and ribosomal RNA genes could be inferred across the phylogeny, direct inference of phylogeny from rearrangement data in MLGO resulted in a highly discordant set of relationships inconsistent with both sequence-based phylogenies and previous morphological analysis. Given the demonstrated rates of genomic evolution within thrips, extensive sampling is needed to fully understand these phenomena across the order.

## Introduction

Complete mitochondrial genomes have been shown to be useful for phylogenetic and evolutionary studies at various taxonomic scales, as they provide more phylogenetic information than individual genes alone^1–4^. The mitochondrial genome of metazoans is typically a circular molecule 14–19 kilobases (kb) in length, that contains a conserved set of 37 genes: 13 protein-coding genes (PCGs), ATPase subunits 6 and 8 (*atp6* and *atp8*), Cytochrome oxidase subunits 1 to 3 (*cox1*-*cox3*), cytochrome b (*cob*), NADH dehydrogenase subunits 1–6 and 4 L (*nad1-6* and *nad4L*); the small and large subunit rRNAs (*rrnL* and *rrnS*), 22 transfer RNA (tRNA) genes, and a non-coding control region (CR) which contains initiation sites for transcription and replication^1,5^. In addition to sequence variation, metazoan mitochondrial genomes also exhibit variation in a number of features, such as length, tRNA secondary structure, gene order, and the number and internal structure of control regions^6–8^. These features can provide evidence for evolutionary relationships among taxa at high and/or low taxonomic levels beyond that provided by analysis of mitochondrial sequence data alone^9–11^.

The thrips, Order Thysanoptera, are a small order of insects, with 6154 described species^12^. The order is currently divided into two suborders, Terebrantia and Tubulifera, and nine families^13^. Despite the modest diversity of the group, the eight published thrips mitochondrial genomes are all from members of the suborder Terebrantia; a single unpublished genome from Tubulifera (GenBank Accession No. KP198620). Even within Terebrantia, information is available for just three of the four subfamilies within a single family, Thripidae: Thripinae (*Anaphothrips obscurus*^14^, *Frankliniella intonsa*^15^, *F*. *occidentalis*^16^, *Scirtothrips dorsalis*^17^, *Thrips imaginis*^18^, and *T*. *palmi*^19^), Dendrothripinae (*Dendrothrips minowai*^20^), and Sericothripinae (*Neohydatothrips samayunkur*^21^).

Multiple classification hypothesis have been proposed for Thysanoptera^13,22–24^, each of which conflicts with studies of thrips phylogenetic relationships. The most widely followed classification of order Thysanoptera recognises two suborders and 13 families (nine extant and four extinct families)^13^. Bhatti elevated Thysanoptera to a superorder with Terebrantia and Tubulifera as orders, and proposed 40 families based on highly conserved structures in the body architecture^24^, including elevating the subfamilies Dendrothripinae, Panchaetothripinae, Sericothripinae, Franklinothripinae to family level. In the only major study of thrips phylogenetics, Buckman *et al*. (2013) used multiple molecular markers to test the relationships within thrips, particularly of families and subfamilies^25^. Their data supported the monophyly of Tubulifera and Terebrantia and of the families Phlaeothripidae, Aeolothripidae, Melanthripidae and Thripidae. However, relationships between the four thripid subfamilies, Dendrothripinae, Sericothripinae, Panchaetothripinae, and Thripinae, were unclear. The two largest subfamilies, Phlaeothripinae and Thripinae, were paraphyletic and require further study to understand their internal relationships. Mitochondrial genome data from Thysanoptera are analysed there in order to further clarify the phylogenetic relationships of the order.

In the present study, four mitochondrial genomes representing three additional thrips families plus the fourth thripid subfamily (Panchaeotothripinae) were sequenced, annotated and compared to the other available thrips genomes. We analysed the main features of the newly generated genomes, including nucleotide composition, secondary structure of tRNAs and control region. To determine relationships within Thysanoptera, analyses with sequence-based and genome rearrangement phylogenetic inference methods were used.

## Materials and Methods

### Ethics statement

Thrips specimens were collected in the field by the beating method. All species used in this study are common on agricultural and horticultural crops and are not listed in “List of Protected Animals in India”. Thus, no prior permission was required for their collection.

### Sample collection and DNA extraction

Specimens of *Franklinothrips vespiformis* (from general vegetation), *G*. *uzeli* (from banyan tree, *Ficus benghalensis*), *H*. *indicus* (from date palm, *Phoenix* sp.) and *R*. *cruentatus* (from pomegranate, *Punica granatum*) were collected from Odisha State, India. Specimens were preserved in absolute alcohol and stored at −80 °C at the Centre for DNA Taxonomy, Molecular Systematics Division, Zoological Survey of India, Kolkata. Morphological identification of all the specimens was done by the author (K.T.) with the help of available taxonomic keys^26–30^. The DNeasy DNA Extraction kit (QIAGEN) was used for the extraction of the genomic DNA following the manufacturer’s standard protocol. DNA Quantity was estimated by using a Qubit fluorometer with the dsDNA high-sensitivity kit (Invitrogen) and by agarose gel (0.8%) electrophoresis.

### Mitochondrial genome sequencing, assembly, annotation

Whole genomic DNA was sequenced using the Illumina NextSeq500 (2 × 150 base paired-end reads) (Illumina, USA) platform. Paired-end libraries were constructed using the TruSeq DNA Library Preparation kit according to standard protocols. Libraries were pooled, cleaned with Highprep magnetic beads (Magbio) and then sequenced on a Nextseq 500, using 2 × 150 chemistry at the Genotypic Technology Pvt. Ltd. Bangalore, India (http://www.genotypic.co.in/). The approximately 25 million raw reads were trimmed to remove the low quality reads by using NGS-Toolkit^31^. Trimmed reads were filtered using the Burrows-Wheeler Alignment (BWA) tool^32^ and then assembled in SPAdes 3.9.0^33^ using default parameters and the *Scirtothrips dorsalis* EA mitochondrial genome as a reference. Aligned reads were used for de novo mitochondrial genome assembly. The annotation of the assembled genome was performed using the MITOS web-server^34^ (http://mitos.bioinf.uni-leipzig.de/index.py) to estimate the location of protein coding (PCGs), transfer RNA (tRNAs), and ribosomal RNA genes (rRNAs). Gene boundaries for PCGs and rRNAs were confirmed manually using BLASTn, BLASTp and ORF Finder (as implemented at NCBI^35^ (https://www.ncbi.nlm.nih.gov/orffinder/). Initiation and termination codons were confirmed in MEGAX^36^ using the published mitochondrial genome sequences of other thrips as references. The secondary structures of tRNAs were predicted by using MITOS, tRNAscan-SE^37^ (http://lowelab.ucsc.edu/tRNAscan-SE/), and ARWEN 1.2^38^. In addition to annotating the four thrips species sequenced here, the mitochondrial genome of *Haplothrips aculeatus* (GenBank KP198620) was re-annotated to identify genes missing from the GenBank record for this species.

### Genome visualization, and comparative analysis

**Figure 1:**
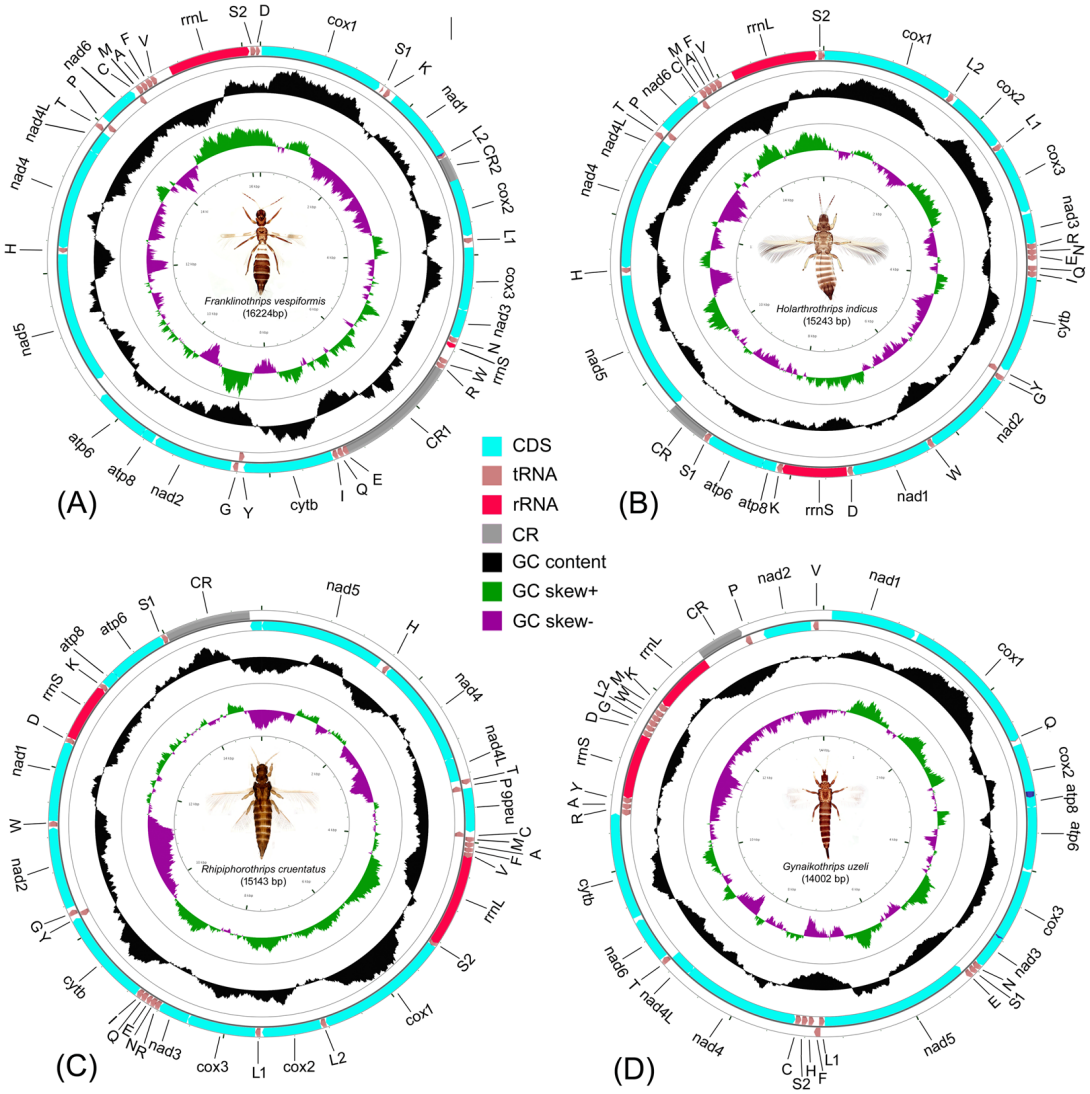
CG view of thrips mitogenomes. (**A**) *F*. *vespiformis*, (**B**) *H*. *indicus*, (**C**) *R*. *cruentatus*, (**D**) *G*. *uzeli*. Different colour arrows are used to show the different genes. Leica Microscope (model DM1000) was used for species images.

The circular genome maps for all four species were predicted by the CGView online server (http://stothard.afns.ualberta.ca/cgview_server/) with default parameters (Fig. 1, Table 1). Differences between tRNA isotypes in thrips species were assessed by p- and maximum composite likelihood distances for each pairwise-comparison using MEGAX^36^. Base mismatches occurring at the boundaries between DHU or TΨC arms and in loop regions were not considered for tRNA distances due to length variation in these areas. The secondary structures within control regions (CRs) were predicted by mfold^39^ (unafold.rna.albany.edu/?q = mfold) using default parameters. Homology between CR copies in thrips species with multiple CRs were determined with a ClustalW^40^ sequence alignment implemented in MEGAX. Tandem repeats within CR were identified with Tandem Repeats Finder^41^ (https://tandem.bu.edu/trf/trf.html).

**Table 1:** Details of newly and previously sequenced mitochondrial genomes of Thysanoptera used in the present study.

Sub-order	Family	Subfamily	Species	Accession No.	Size (bp)	Reference
Terebrantia	Aeolothripidae		*Franklinothrips vespiformis*	MN072395	16,224	This Study
	Stenurothripidae		*Holarthothrips indicus*	MN072397	15,243	This Study
	Thripidae	Dendrothripinae	*Dendrothrips minowai*	MF582634	14,631	Chen SC, *et al*.^20^
		Panchaetothripinae	*Rhiphiphorothrips cruentatus*	MN072396	15,143	This Study
		Sericothripinae	*Neohydatothrips samayunkur*	MF991901	15,295	Kumar *et al*.^36^
		Thripinae	*Thrips palmi*	MF991901	15,333	Chakraborty *et al*.^19^
			*Anaphothrips obscurus*	KY498001	14,890	Liu *et al*. 2017
			*Scirtothrips dorsalis* EA	KM349826	15,343	Dickey *et al*.^17^
			*Scirtothrips dorsalis* SA	KM349827 & KM349828	15,204	Dickey *et al*.^17^
			*Frankliniella intonsa*	JQ917403	15,215	Yan *et al*.^15^
			*Frankliniella occidentalis*	JN835456	14,889	Yan *et al*.^16^
			*Thrips imaginis*	AF335993	15,407	Shao *et al*. 2001
Tubulifera	Phlaeothripidae	Phlaeothripinae	*Gynaikothrips uzeli*	MK940484	14,002	This Study
			*Haplothrips aculeatus*	KP198620	14,616	Unpublished

### Data set preparation, model selection and phylogenetic analysis

Phylogenetic analysis was conducted on the PCGs from the 10 thrips mitochondrial genomes, the four species newly sequenced here and a representative of the Hemiptera (*Alloerhunchus bakeri*)^42^ as an outgroup. This outgroup was chosen as Hemiptera are generally considered to be the sister group of thrips^43^. Sequences for each PCG were aligned individually with codon-based multiple alignments using MAFFT as implemented in the TranslatorX^44^. Given that initial alignments resulted in some ‘gappy’ and potentially poorly aligned regions, masking of ambiguously aligned sites was performed using GBlocks^45^ (as implemented in TranslatorX) using default setting. Ambiguously aligned sites were removed from the protein alignment before back-translation to nucleotides. The dataset of all PCGs were concatenated using SequenceMatrix v1.7.845^46^. To assess the impact of high substitution rates and potentially poorly aligned regions within genes, four data sets were constructed: (1) all 13 PCGs, all codon positions, 12334 bp; (2) all 13 PCGs, third codon position excluded, 8124 bp.: (3) all 13 PCGs, all codon positions, masked with GBlock,s 8421 bp; (4) all 13 PCGs, third codon position exluded, masked with GBlocks, 5614 bp. To find the best substitution models, PartitionFinder version 2.1.1^47^ was used with the ‘greedy’ algorithm and the following predefined partitions: codon positions for each PCG (39 partitions for datasets 1 and 3, 26 for datasets 2 and 4) (Supplementary Table S1). The CIPRES Science Gateway v.3.1^48^ (www.phylo.org/sub_sections/portal/) portal was used to infer both Bayesian (BI) and maximum likelihood (ML) phylogenies for each of the 4 datasets. BI analyses were conducted with Mr.Bayes ver. 3.2^49^ with two MCMC runs each with four chains (three heated and one cold) run for 500,000 generations, with tree sampling every 100 generations and a burn-in of 25%. The ML analysis was performed using IQ tree ver.1.6.10^50,51^ with 1000 replicates of ultrafast likelihood bootstrap to obtain a consensus tree. Phylogenetic trees were visualized and edited using FigTree ver 1.4.2^52^ (http://tree.bio.ed.ac.uk/software/figtree/).

### Gene order analysis and ancestral state estimation

Three methods were applied to estimate gene arrangement history in Thysanoptera: (1) mapping of ancestral and shared derived boundaries; (2) common interval analysis; and (3) maximum likelihood analysis of gene order. The vast majority of insect mitochondrial genomes sequenced to date share an identical gene order, the inferred ancestral insect mitochondrial genome^1^, which is most parsimoniously inferred to have been present in the common ancestors of many insect orders including the sister-group of thrips, Hemiptera^53^. While mitochondrial genome rearrangements have been found in some hemipteran species, in each instance these are confined to derived clades representing disparate groups. All mitochondrial gene rearrangements observed in thrips, thus occurred at some point after the common ancestor of Thysanoptera + Hemiptera, and the evolutionary pattern of these rearrangements is inferred relative to this ancestor who retained the inferred ancestral insect mitochondrial genome^1^.

Gene boundary mapping was conducted by pairwise comparison of each thrips genome to the inferred ancestral insect mitochondrial genome^1^ to determine the retention of ancestral and derived gene boundaries following Yoshizawa *et al*.^54^ (Supplementary Table S2). The longest series of shared gene-boundaries between any two species were identified as ‘gene blocks’ and species with only a portion of the gene block (i.e. 1 or more of its gene boundaries) identified as possessing a ‘modified gene block’. Shared ancestral and derived gene boundaries/blocks were mapped onto the BI tree inferred from dataset 1.

Common interval analysis was conducted using CREx^55^ for pairwise comparisons and TreeREx^56^ for inference of ancestral genome states. Pairwise comparisons using CREx were performed for each of the four newly sequenced thrips genomes against the inferred ancestral insect mitochondrial genome to determine the minimum number of genome rearrangement events separating each thrips species from the ancestral state. CREx considers four types of rearrangement events: transpositions, inversions (gene inverted but not transposed), inverse transpositions (gene both inverted and transposed) and tandem-duplication/random loss (TDRL) involving the duplication and rearrangement of multiple genes within a single gene block.

By comparing genome common intervals over a fixed phylogenetic tree, TreeREx^56^ infers genome states for the common ancestor at each node in that tree, and allows the order of genome rearrangements in evolutionary time to be inferred. TreeREx utilises the same common interval algorithm as CREx and the same set of rearrangement events (transpositions, inversions, inverse transpositions, TDRL). TreeREx analysis was performed with default settings: strong consistency method applied (-s); weak consistency method applied (-w); parsimonious weak consistency method applied (-W); obtain alternative bp scenario for prime nodes (-o); maximum number of inversions (−m = 0), +TDRL scenarios considered.

As both CREx and TreeREx are incapable of handling genomes without fixed gene sets, both missing data and the duplication of genes as observed in thrips violates the algorithm and require work arounds. Genes that were apparently missing (one tRNA), were omitted from pairwise CREx analyses for the species in which they were not detected (Supplementary Table S3). TreeREx analyses of all thrips taxa were conducted using just the relative order of the major genes (PCGs, rRNAs, primary CR) because some species were missing tRNA genes and others had duplicate copies of tRNAs (Supplementary Table S4). Preliminary analyses eliminating both missing and duplicated genes from all species but retaining all other tRNAs indicated such high rates of tRNA rearrangement that any phylogenetic patterns in the remaining genes could not be identified. Treating tRNA rearrangements as ‘noise’ in such reconstructions has been previously applied to identify ancestral genome arrangements for each of the bilaterian superphyla^34^, and is an efficient way to account for the differences in rearrangement rate between classes of mitochondrial genes. This approach was taken here.

MLGO (maximum likelihood analysis of gene order)^57^ is an algorithm for directly inferring phylogeny based on gene order data, and is not limited to a common gene set across taxa in the way that CREx/TreeREx is. Complete gene order data, including duplicated and excluding missing genes only from those taxa in which they were undetected were used to infer a phylogeny of thrips (Supplementary Table S5). The MLGO phylogeny was compared against those generated from sequence data alone (datasets 1–4, both BI and ML analyses) to determine the direct phylogenetic signal from gene-order data alone.

## Results

### Genome structure, organization and composition

We sequenced complete mitochondrial genomes of *F*. *vespiformis* (16,224 bp long), *H*. *indicus* (15,243 bp), *R*. *cruentatus* (15,143 bp) and *G*. *uzeli* (14,002 bp) (Fig. 1, Supplementary Table S6). All four species had at least the canonical 37 genes found in most Metazoa, except *G*. *uzeli* in which *tRNA* isoleucine (*trnI*) was not detected. One control region (CR) was detected in *H*. *indicus*, *R*. *cruentatus* and *G*. *uzeli* while two were found in *F*. *vespiformis*.

Most of the genes in *F*. *vespiformis*, *H*. *indicus*, and *R*. *cruentatus* were encoded on the majority strand except three PCGs (*nad5*, *nad4*, *nad4L*) and three tRNAs (*trnC*, *trnH* and *trnP*). Although the majority of genes in *G*. *uzeli* were also encoded by the majority strand, a slightly larger number were encoded on the minority strand, four PCGs (*nad5*, *nad4*, *nad4L*, *nad2*), fifteen tRNAs (*trnL1*, *trnH*, *trnS2*, *trnC*, *trnR*, *trnA*, *trnY*, *trnG*, *trnD*, *trnL2*, *trnW*, *trnM*, *trnK*, *trnP*, *trnV*) and both ribosomal RNAs (*rrnS*, *rrnL*). All four genomes were AT rich (*F*. *vespifomis*: 75.35%, *H*. *indicus*: 74.89%, *R*. *cruentatus*: 76.58%, and *G*. *uzeli*: 82.15%) as has been observed in other thrips and almost all insect mitochondrial genomes (Supplementary Table S7).

### Transfer RNAs

All fourteen of the thrips mitochondrial genomes that we examined had the 22 tRNAs found in the ancestral insect mitochondrial genome except for *G*. *uzeli*, in which *trnI* was not detected. Duplicated tRNAs were detected in *H*. *aculeatus* (*trnM*) and in *T*. *imaginis* (*trnS1*, *trnE*). In *H*. *aculeatus*, the second copy of *trnM* (located between *trnV* and *trnL2*) is an exact duplicate of the first copy (65 bp, between *trnK* and *trnV*) with same anticodon (CAT), so it is difficult to predict which copy, or both, is functional. In *T*. *imaginis*, the first copy of *trnS1* (between *trnD* and *trnL1*) is 55 bp long whereas the second copy (between CR2 and *trnP*) is 69 bp and 33.3% similar to the first copy; both have an NCT anticodon. Previous analysis suggested that the *trnS1* copy that lies between *trnD* and *trnL1* is more similar to copies of this gene in other thrips species than is the second copy (between CR2 and *trnP*)^18^. In *T*. *imaginis*, the first copy of *trnE* (between *trnN* and CR2) is 64 bp whereas the second copy (between *trnD* and *trnL1*) is 29 bp and is 45.3% similar to the first copy; the first copy is clearly the functional one as the second copy lacks an anticodon arm.

A comparison of tRNA secondary structure and nucleotide substitutions across thrips mitochondrial genomes that we examined are provided (Supplementary Figs. S1 and S2). In most thrips sequenced to date tRNAs have the typical cloverleaf secondary structure with a few exceptions. The DHU stem and loop are absent from *trnS1* in all thrips species except *H*. *indicus*, *H*. *aculaetus*, *F*. *intonsa*, and *F*. *occidentalis*; from *trnV* in all species except *F*. *vespiformis*, *G*. *uzeli*, *H*. *indicus*, *H*. *aculaetus*, and *D*. *minowai*; and from *trnN* in *A*. *obscurus*. The TΨC stem and loop is absent from *trnL2* in *G*. *uzeli*, from *trnT* in *N*. *samayunkur* and *T*. *palmi*, and from *trnR* in *N*. *samayunkur*. Lack of the DHU stem-loop in *trnS1* is nearly ubiquitous in insect mitochondrial genomes, however the absence of this or other stem-loops from other tRNA isotypes is extremely uncommon^58^. Finally, there is a consistent difference in anti-codon sequence for *trnA* between the thrips suborders: GCT in Terebrantia versus GCA in Tubulifera. Anticodon loop mutations, even in the ‘wobble’ nucleotide are comparatively uncommon in insects generally.

Calculations of the p-distance (pDis), maximum- likelihood distance (MLdis) and base difference (BDps) showed that the *trnE* is the most conserved tRNA (BDps = 6.363, MLdis = 0.293 ± 0.18 and pDis = 0.163 ± 0.07), whereas *trnD* is the most variable (BDps = 19.04, MLdis = 0.592 ± 0.24 and pDis = 0.359 ± 0.09). *trnI* was not analysed for substitution patterns as it was not detected in *G*. *uzeli* (Supplementary Table S8).

### Control regions

**Figure 2:**
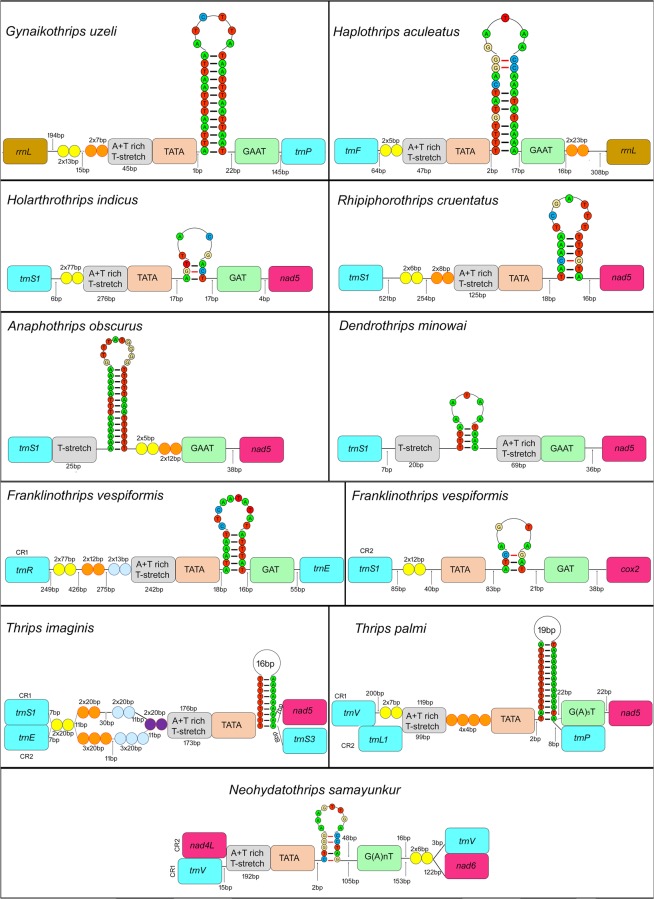
Control region of *G*. *uzeli*, *H*. *aculeatus*, *H*. *indicus*, *R*. *cruentatus*, *A*. *sudanensis*, *D*. *minowai*, *F*. *vespiformis*, *T*. *imaginis*, *T*. *palmi*, *N*. *samayunkur*. Different colours were used to show the structural elements.

**Figure 3:**
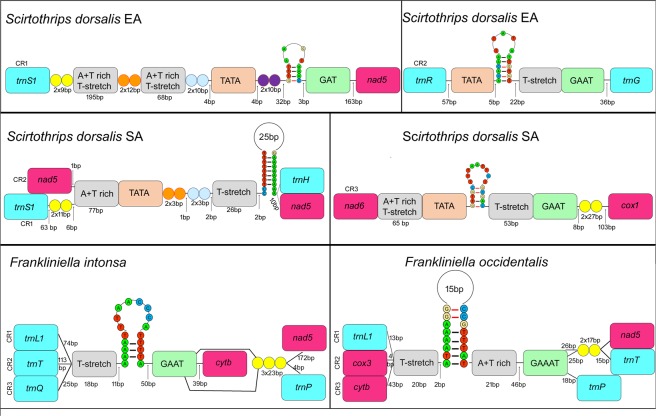
Control region of *S*. *dorsalis* EA and SA, *F*. *intonsa*, *F*. *occidentalis*. Different colours were used to show the structural elements.

The control region is typically the largest non-coding region in the mitochondrial genome, and is heavily biased to A + T nucleotides. The CRs of thrips vary in number, size, and location within the genome across the 13 species sequenced to date due to duplications and gene rearrangements. Three control regions are observed in the *F*. *intonsa*, *F*. *occidentalis*, and *S*. *dorsalis* SA, two in *F*. *vespiformis*, *N*. *samayunkur*, *S*. *dorsalis* EA, *T*. *imaginis*, and *T*. *palmi*, while the remaining 6 thrips species have only one control region (Figs. 2 and 3). Conserved motifs (including a poly T-stretch at the 5′ end, TA(A)n-like stretch, hairpin loop structures, TATA motif, and G(A)nT motif) within the CR have been identified as initiation sites for replication and transcription^6,59–61^. These conserved motifs are also detected in thrips, however the arrangement of motifs differs between species.

Previous studies have shown a conserved location of at least one CR between *atp6* and *nad5*, as it was found in all species from the family Thripidae except *Neohydatothrips* (variable sets of tRNAs are also found between the CR and *atp6* in different species, but *atp6* is the closest major upstream major gene)^14^. This CR was thus proposed to be the original location for Thysanoptera as a whole, despite the absence of data from families other than Thripidae, and that this region was homologous between species that had multiple CRs. However, with our expanded sampling it is clear that this conserved position for the CR is confined to Thripidae + Stenurothripidae, whereas CR location differs in Aeolothripidae (between *cytb* and *rrnS*), and in each of the two Phlaeothripidae examined between *rrnL* and *nad2* (in *Gynaikothrips*) and between *rrnL* and *atp6* (in *Haplothrips*). The position of both CRs within *Neohydatothrips*, between *nad4L* and *nad6*, appears to be secondarily derived, differing as it does from all other thripids sampled to date.

Five thrips species (*F*. *vespiformis*, *N*. *samayunkur*, *S*. *dorsalis* EA, *T*. *imaginis*, and *T*. *palmi*) have two CRs (Figs. 2 and 3). Sequence similarity between CRs varies widely across species from as low as 10.03% in *F*. *vespiformis* to 99% in *T*. *imaginis*. Species with higher similarity between CRs correspondingly have higher similarity in the makeup of their CR motifs, i.e. *T*. *imaginis* CR motifs are almost identical, while *N*. *samayunkur* (44.2% similar) CRs share some features: two tandem repeats of 6 base pairs, 192 bp A + T rich and T-stretch region following with TATA motif, 18 bp hairpin loop flanking with G(A)nT.

A further three thrips species (*F*. *intonsa*, *F*. *occidentalis*, and *S*. *dorsalis* SA) possess three putative CRs (Fig. 3). The CR1 of *F*. *intonsa* (between *atp6* and *nad5*, the putative ancestral location in Thripidae) has 54% sequence similarity with CR2 and 50% to CR3, whereas CR2 and CR3 show 91.15% sequence similarity to each other. Each of the three *F*. *intonsa* CRs share an 18 bp poly T-stretch, an 18 bp hairpin loop structure, the GAAT motif, whereas three 23 bp long tandem repeats (TRs) are present in CR1 and CR3. *F*. *occidentalis* has three CRs in the same genomic locations as its congener *F*. *intonsa*, and has a similar pattern of sequence similarity: CR1 is 89.15% similar to CR2 and 86.32% to CR3, while CR2 and CR3 show 98.92% sequence similarity to each other. The three CRs in *F*. *occidentalis* also share poly T-stretches (20 bp), hairpin loops (31 bp), A + T rich region (21 bp) and GAAAT motifs, while two of them (CR1 and CR2) share TRs of 17 bp. While the South Asia isolate of *Scirothrips dorsalis* has three mitochondrial CRs, comparisons are complicated by the fact that this isolate has a multipartite genome, with two CRs on the larger chromosome (representing 93% of genome length, 35/37 genes), and a third on the second, smaller chromosome (representing the remaining 7% and 2/37 genes)^14^. The CR1 of *S*. *dorsalis* SA (again between *atp6* and *nad5*) was 51.74% similar to CR2 and 34.88% to CR3, while CR2 and CR3 showed 49.1% sequence similarity to each other. The two CRs on the larger chromosome (CR1 and CR2) possess a 77 bp A + T rich region, TATA motif, two classes of 3 bp TRs (GAA and GTA), 26 bp poly T-stretch, and a 45 bp hairpin loop structure without the GAT motif. In contrast, CR3 possess all the conserved CR motifs plus two TRs of 27 bp each.

### Phylogenetic relationships

**Figure 4:**
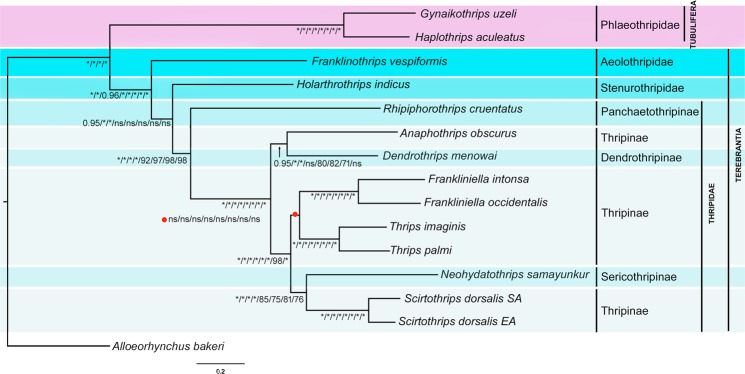
Phylogenetic tree (BI-1): support values for BI and ML trees are in the following order (BI-1/BI-2/BI-3/BI-4/ML-1/ML-2/ML-3/ML-4). The posterior probabilities (1.0) and bootstrap support (100%) are represented with an asterisk. The values, posterior probabilities below (0.9–1.0) and bootstrap support (70–100%) are shown by ‘ns’.

We generated eight phylogenetic trees based on four datasets and two inference methods (BI and ML). The topology most commonly recovered across this study is represented by analysis BI-1 (Fig. 4), and variation between analyses is reported below. The suborders Tubulifera and Terebrantia were recovered as monophyletic in all analyses. In six analyses (BI-1 to 4 and ML-1 and ML-3) Stenurothripidae is sister to Thripidae, whereas, in analyses ML-2 and ML-4 (datasets which exclude third codon positions) Aeolothripidae is sister to Stenurothripidae but with low bootstrap support. Thripidae is monophyletic in all analyses, with the subfamily Panchaetothripinae sister to the remaining three subfamilies (Dendrothripinae, Sericothripinae and Panchaetothripinae) in all analyses (Supplementary Figs. S3–S6). Dendrothripinae was sister to part of the Thripinae (*Anaphothrips*) in the majority of analyses (BI-1, 2, 4, ML-1, 2), but was sister to a clade composed of Thripinae and Sericothripinae species in analyses BI-3, ML-3 and ML-4 trees. The subfamily Sericothripinae always grouped with *Scirtothrips*, rendering Thripinae paraphyletic in all trees. The genera *Frankliniella*, *Thrips* and *Scirtothrips* were monophyletic in all analyses, however relationships between Thripinae genera varied between analyses: *Frankliniella* + *Thrips* (BI-1, 2, 4 and ML-3, 4) versus *Frankliniella* + (*Neohydatothrips* + *Scirtothrips*) (BI-3, ML-1, 2). Genus level relationships appear quite sensitive to inclusion/exclusion decisions regarding potentially high variability sites (both third codon positions and GBlocks masking) and had low nodal support.

### Mapping shared gene orders

**Figure 5:**
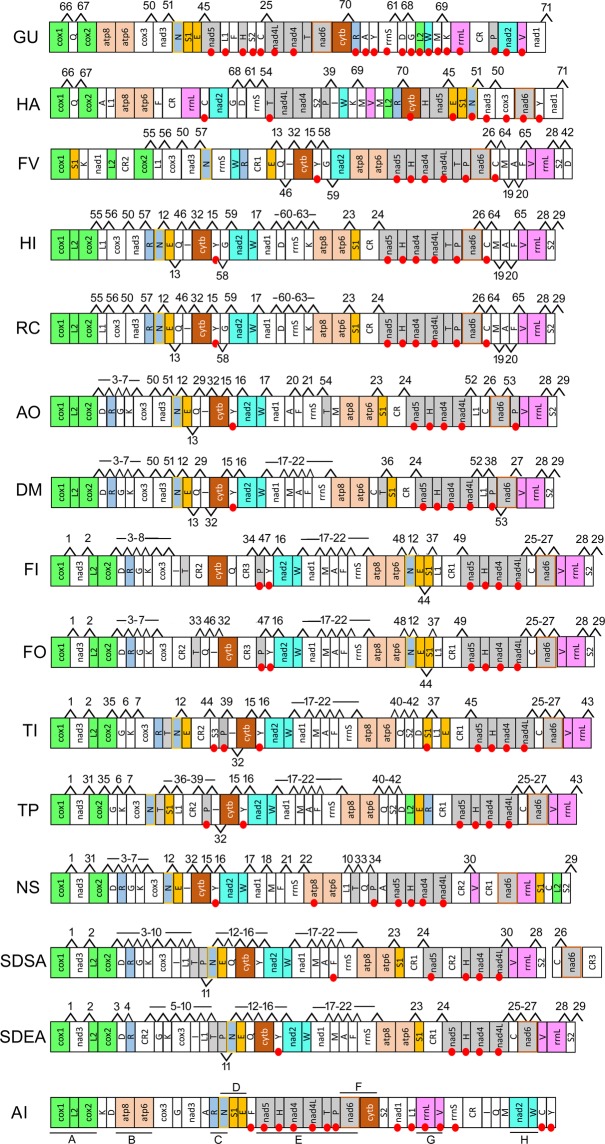
Gene order representation. Gene blocks with red circles show the position of genes on the minority strand. Ancestral gene blocks A-H are underline in the *A*. *bakeri* gene order and also indicated by different colours. Different codes were used to label the boundaries.

**Figure 6:**
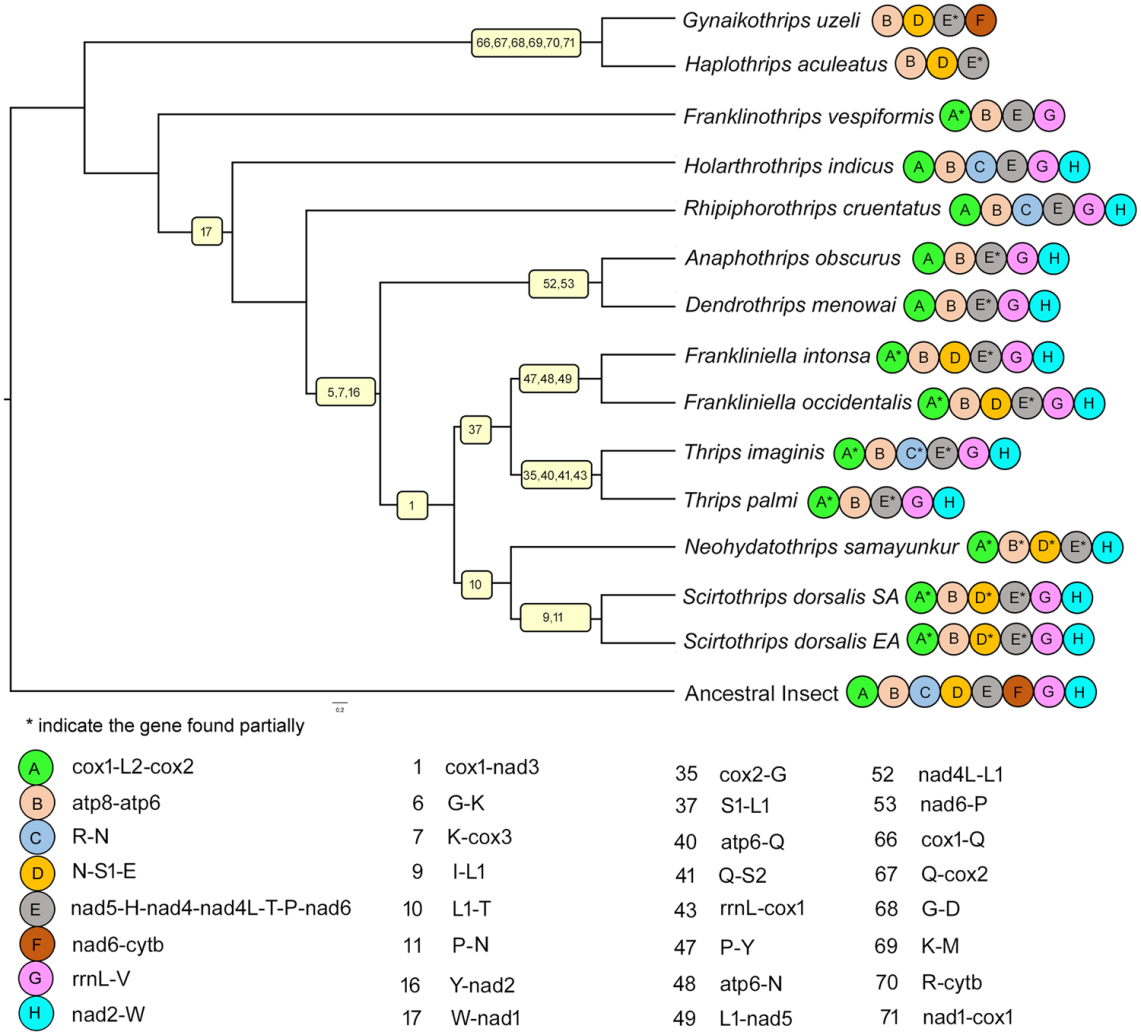
Representation of derived characters on phylogenetic tree. BI-1 is used for representation of the ancestral and shared derived characters. Shared derived character states are shown on the node. Ancestral gene blocks (**A**–**H**) are shown with different colours and code, shown at the terminal end of the branch. Partial ancestral characters are marked with an asterisk.

Linearised gene order maps for 14 thrips mitochondrial genomes plus the inferred ancestral insect gene order are given in Fig. 5. Eight conserved gene blocks (labelled A-H) that have been retained from the ancestral insect in at least one thrips species are mapped on the gene orders (Supplementary Table S2). These ancestral gene blocks consist of between 2 and 7 genes (1–6 gene boundaries) and 150–4000 bp of the genome. Gene blocks that are partly conserved (either due to loss of a portion of the genes or the translocation of other genes into the middle of the gene block) are also noted. Gene block A (*cox1-trnL2*-*cox2*) is conserved in *H*. *indicus*, *R*. *cruentatus*, *D*. *minowai*, *A*. *obscurus*, and partially present in most of the remaining Thripidae species (*nad3* is inserted in *Frankliniella*, *Scirtothrips* and *T*. *imaginis*). Gene block B (*atp8*-*atp6*) is retained in all thrips species except *N*. *samayunkur* where *atp8* gene has been inverted. Block C (*trnR*-*trnN*) is conserved only in *H*. *indicus* and *R*. *cruentatus*, and partially present in *T*. *imaginis* (*trnT* is inserted between *trnR*-*trnN*). Block D (*trnN*-*trnS1*-*trnE*) is conserved in Phlaeothripidae, while a shuffled version of this gene block (*trnN*-*trnE*-*trnS1*) occurs in species of *Frankliniella*. In contrast, *trnS1* is translocated from gene block D in *Scirtothrips* species and *N*. *samayunkur*. Block E (*nad5*-*trnH*-*nad4*-*nad4L*-*trnT*-*trnP*-*nad6*) is conserved in *H*. *indicus*, *R*. *cruentatus* and *F*. *vespiformis*. Other thrips species have either portions of this gene block (*nad5* to *nad4L* in 8 species) or minor variations due to translocation of tRNAs or CRs into this gene block (3 species). In Phlaeothripidae, block E is split into two gene blocks (*nad5*-*trnH*) and (*nad4*-*nad4L-trnT*). Block F (*nad6*-*cytb*) is found only in *G*. *uzeli*. Block G (*rrnL*-*trnV*) is conserved in all Terebrantia species except *N*. *samayunkur*. Block H (*nad2*-*trnW*) is similarly conserved in Terebrantia except *F*. *vespiformis*. The phylogenetic distribution of the ancestral gene blocks is shown on Fig. 6.

Four hundred and three derived gene boundaries are found in the 14 available mitochondrial genomes of thrips. Of these, 71 are shared, derived gene boundaries (numbered 1 to 71 in Fig. 5) present in at least two species and a further 90 unique boundaries found in a single species were identified. The gene order of *H*. *indicus* (Stenurothripidae) is identical to that of *R*. *cruentatus* (Thripidae) sharing 25 derived gene boundaries. Four of these 25 gene boundries (57, 60, 62, 63) are exclusive to these two species, while six (55, 56, 58, 59, 64, 65) are shared with *F*. *vespiformis* and one (61) is shared with tubuliferans.

Out of the 71 derived gene boundries, 47 are either homoplastic or secondarily lost in some of the taxa descended from the node and remaining 24 are unambiguously synapomorphies. Hence, 24 shared derived gene boundaries were mapped onto the phylogenetic tree inferred from the BI-1 dataset (Fig. 6). Derived gene boundary 17 (*trnW*-*nad1*) is a synapomorphy for all terebrantians except Aeolothripidae, consistent with the majority of the phylogenetic analyses (BI-1 to 4, ML-1, 3). Gene boundaries 6, 7 and 16 (*trnG*-*trnK*, *trnK*-*cox3*, and *trnY-nad2*) are synapomorphies for the family Thripidae exclusive of subfamily Panchaetothripinae. Gene boundaries 52 and 53 (*nad4L*-*trnL1* and *nad6*-*trnP*) are synapomorphic for the *A*. *obscurus* and *D*. *minowai* clade, again consistent with the majority of phylogenetic analyses (BI-1, 2, 4, ML-1, 2). Gene boundary 1 (*cox1*-*nad3*) is a synapomorphy for the clade composed of ‘thripinae’ i.e. all genera other than *Anaphothrips*. Gene boundary 37 (*trnS1*-*trnL1*) is synapomorphic for the clade *Frankliniella* plus *Thrips*. Gene boundary 10 (*trnL1*-*trnT*) is a synapomorphy for *Neohydatothrips* + *Scirtothrips*. Gene boundaries 47, 48, and 49 (*trnP*-*trnY*, *atp6*-*trnN* and *trnL1*-*nad5*) are synapomorphies for the genus *Frankliniella* whereas boundaries 35, 40, 41, and 43 (*cox2*-*trnG*, *atp6*-*trnQ*, *trnQ*-*trnS2*, *rrnL*-*cox1*) are synapomorphies for *Thrips*. Gene boundary 9 (*trnI*-*trnL1*) and 11 (*trnP*-*trnN*) are synapomorhies for *Scirtothrips* again consistent with the majority of phylogenetic analyses (BI-1, 2, 4, ML-1, 2). The species of Tubulifera have six (66 to 71) synapomorphic gene boundaries (*cox*-*trnQ*, *trnQ*-*cox2*, *trnG*-*trnD*, *trnK*-*trnM*, *trnR*-*cytb*, *nad1*-*cox1*).

### Common-interval analysis of gene order with CREX

#### Gene rearrangements in *F*. *vespiformis*

Two alternative scenarios, each with seven rearrangement events were inferred: transposition of *trnI*, inversion of two gene blocks three major genes (*nad1*, *trnL1*, *rrnS*, *trnV*, *rrnL* and *trnF*) and four TDRL events. In total, six PCGs (*nad3*, *cytb*, *nad2*, *nad1*, *atp8*, *atp6*) were rearranged relative to their position in the ancestral genome. Most tRNAs were in derived positions, and a second CR separates the genes *trnL2* and *cox2* which are otherwise a conserved gene boundary (part of block A) (Supplementary Fig. S7).

#### Gene rearrangements in *H*. *indicus*, and *R*. *cruentatus*

The gene order of *R*. *cruentatus* is identical to that of *H*. *indicus* despite these two taxa belonging to different families. Again, two alternative scenarios each with seven events were inferred, including the same transposition and inversions as in *F*. *vespiformis* with a different set of 4 TDRL events. Most of the major genes and tRNAs are rearranged relative to the ancestral genome (Supplementary Fig. S7).

#### Gene rearrangements in *G*. *uzeli*

Two alternative scenarios, each consisting of 12 inversions and 5 TDRL events were inferred. Twelve of the 15 major genes are in the same relative positions as in the ancestral gene order (*nad4/nad4L* and *rrnL/rrnS* have been shuffled in gene order, while *nad1* is between *nad2* and *cox1*). Two major genes (*nad1* and *nad2*), and ten tRNAs (*trnF*, *trnS2*, *trnR*, *trnA*, *trnD*, *trnG*, *trnL2*, *trnW*, *trnM*, *trnK*) have been inverted (Supplementary Fig. S8).

### Reconstructing genome evolution across the thrips phylogeny

Due to very high rates of tRNA rearrangement (see preceding section), the relative gene order of the major genes (PCGs and rRNAs) were used to infer the gene order of the common ancestors at each node in the phylogeny (Supplementary Table S4). Replicate analyses were run in TreeREx of the tree inferred from the BI-1 and ML-3 datasets as these represented the most topologically divergent phylogenetic trees recovered from sequence data. The differences between these two topologies, however, did not involve taxa whose relative gene orders differed. For instance, different resolutions of the relationships between the genera *Frankliniella*, *Thrips*, *Neohydatothrips* and *Scirtothrips* are uninformative with respect to gene order, as each of these gene all share the same arrangement of their major genes.

**Figure 7:**
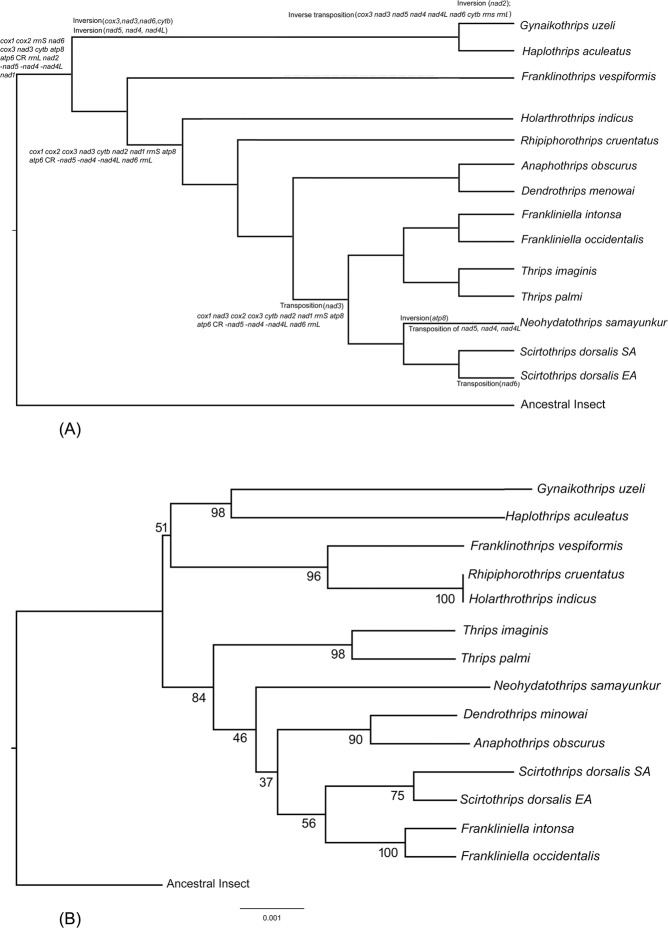
(**A**) Original output of the TreeREx analysis. The rearrangements on the branches are given as Transposition, Inversion, Inverse transposition and Tandem-Duplication-Random-Loss events (TDRLs). (**B**) MLGO tree.

Large-scale genome rearrangement through TDRL events that duplicated most of the genome were inferred to have occurred between the common ancestor of Thysanoptera and the ancestral insect gene order (Fig. 7A, Supplementary Table S9). Subsequent, similarly large-scale TDRL events were inferred between the common ancestor of Thysanoptera and the Tubulifera, and between the common ancestor of Terebrantia and *Franklinothrips*. Modest rearrangements (e.g., transposition) of *nad3* in the common ancestor of the clade composed of *Frankliniella*, *Thrips*, *Neohydatothrips* and *Scirtothrips*, and in some terminal taxa e.g. *Neohydatothrips* and *Scirtothrips dorsalis* SA, were found in the more derived portions of the tree.

### Genome order analysis

#### Inferring thysanoptera phylogeny directly from gene-order

The MLGO phylogeny is based on the complete gene-order data including duplicated genes, and excluding missing/unannotated genes on a species-by-species basis as appropriate depending on their original genome annotations. The MLGO topology is highly discordant with all the phylogenies inferred from sequence datasets (both BI and ML analysis, and all 4 datasets) (Supplementary Table S6, Fig. 7B). In the MLGO phylogeny Tubulifera renders Terebrantia paraphyletic, as a clade composed of Aeolothripidae, Stenurothripidae and subfamily Panchaeothripinae was found to be sister to Tubulifera. Relationships within the remaining Thripidae were both inconsistent with previous taxonomic classifications (i.e. Thripinae was paraphyletic) and with the sequence-based phylogeny. While the clade *Anaphothrips* + *Dendrothrips* and genus-level monophyly of *Thrips*, *Frankliniella* and *Scirtothrips* was supported by both gene-order and sequence based phylogenies, relationships between the groups varied greatly between the data types. Broadly speaking the MLGO phylogeny appears to group taxa by the extent of gene rearrangements rather than shared gene orders. For instance, the clade of Tubulifera, Aeolothripidae, Stenurothripidae and Panchaeothripinae includes all the taxa with the most divergent thrips mitochondrial genomes, whereas the other clade composed of Thripidae (excluding Panchaeothripinae) have broadly similar gene orders (c.f. Fig. 7A).

## Discussion

The additional mitochondrial genomes sequenced from thrips for this study greatly extend our understanding of their evolution within Thysanoptera. All but one of the previously available genomes, and all published analyses of thrips mitochondrial genomes, have been confined to the family Thripidae, the second-most speciose thrips family and which includes the most agricultural pest species^12^. In expanding the taxonomic scale of available thrips mitochondrial genomes (three additional families, one additional thripid subfamily) we have greatly expanded our understanding of the evolutionary dynamics of the mitochondrial genome within the order and have enhanced our knowledge of the thrips phylogeny.

### Evolution of mitochondrial genome architectures in thrips

The additional species sequenced in the present study demonstrate the absence of a predictable taxonomic scale for the evolution of mitochondrial gene order and genome architecture in Thysanoptera. Gene order is identical between members of two different families (Stenurothripidae and Panchaeothripinae), yet differs between congeneric species (e.g. *T*. *imaginis* vs. *T*. *palmi* and *F*. *intonsa* vs. *F*. *occidentalis*). Similarly, some derived mitochondrial genome structures are conserved across large clades within thrips, while other derived architectures differ between populations within a single species. For example, multiple CRs, which are otherwise rare within insects^1^, are found in all members of the clade comprising most of the thripine genera plus *Neohydatothrips*, whereas genome fragmentation varies between populations of *Scirtothrips dorsalis*^17^. The taxonomic scale of derived mitochondrial genomic features observed in thrips thus cannot be assumed based on limited sampling. For example, our additional sampling revealed that genomic features previously ascribed to the order Thysanoptera, such as multiple CRs being typical for the order^16,20^, are rather only features of the family Thripidae. Although we are able to identify new trends in mitochondrial genomic evolution in thrips, and refine our understanding of other trends on the basis of extra sampling, considerable additional study is required to verify these proposals.

Expanded sampling supports earlier proposals that tRNAs are subject to higher rates of evolutionary change than are observed in either the PCGs or the rRNAs, including gene duplication, loss, secondary structure variation, anti-codon mutations and rearrangements. Duplicated tRNAs have now been identified from multiple, distantly related thrips species, including representatives of both suborders (*Haplothrips* in Tubulifera and *Thrips imaginis* in Terebrantia). These duplicated tRNAs include both examples where duplicate tRNAs are sequence identical (*trnM* in *Haplothrips*) and ones which show significant sequence divergence (*trnS1* and *trnE* in *T*. *imaginis*). Across insects, sequence identical tRNA duplications are more common, typically in the form of tandem-repeats (e.g. in Hymenoptera^62^). However, the example in *Haplothrips* is not a tandem-repeat (*trnV* separates the two copies). Both sequence-identical and diverged duplicate copies of genes are predicted intermediary products of the tandem-duplication/random-loss (TDRL) model of gene rearrangements (being present between the duplication step and later complete loss of one of the gene copies)^63^, however they are rarely observed in insects relative to completed rearrangements^1^. Indeed, within thrips multiple, almost complete genome length, TDRL events were inferred from the CREx analysis, with 90 derived gene-boundaries (most of which involve tRNA rearrangements) found by comparative genome mapping, yet duplicated tRNAs were found in just two species. This suggests that even in genomically ‘active’ groups such as thrips, TDRL events must resolve quite rapidly in evolutionary time for us to observe so few species with intermediate TDRL products such as duplicate genes. Similar conclusions have been drawn from analyses of Hymenoptera, another insect order with rapid rates of tRNA rearrangement^64^.

Similarly, expanded taxonomic sampling sheds further light on the phenomena of multiple control regions (CRs) in thrips. Multiple CRs are a rare feature within insect mitochondrial genomes with the majority of examples coming from taxa with fragmented genomes such as lice^65^, where each chromosome has one CR. Multiple CRs within a single mitochondrial chromosome, as in thrips, are very rare in insects or other arthropods (e.g. ticks^66^) but somewhat more commonin vertebrates, especially birds^67^. Previous authors have proposed that the presence of multiple CRs in thrips may increase the rate of gene rearrangement^18^. The expanded sampling in the present study suggests that this is not the case. Within Thripidae, there is a limited degree of rearrangement between species with a single CR (*Rhipiphorothrips*, *Anaphothrips* and *Dendrothrips*) and those in the main ‘Thripinae’ clade (including Sericothripinae but excluding *Anaphothrips*), all of whose members have multiple CRs (several tRNA genes and the synapomorphic translocation of *nad3* to between *cox1* and *cox2* in the later clade). Conversely, there are few rearrangements between congeneric species which both possess multiple CRs (e.g. *Thrips* and *Frankliniella*), while in *Scirtothrips* while the two studied population possesses a duplicate CR in different locations in the mitochondrial genome, they differ by no other gene rearrangements^17^. Taken together these features suggest that the interaction between CR duplication and gene rearrangement is not causal. At best CR duplication is likely part of large TDRL events (such as that which translocated *nad3* in the main ‘Thripinae’ clade), not a genomic feature which could drive ongoing rearrangement once introduced into a genome^18^. Thrips species with multiple CRs vary widely in the degree of sequence conservation between CR copies, from very high sequence identities between CRs in *Thrips* and *Frankliniella* spp. (>90%) to as low as 10% identity in *Franklinothrips*. Concerted evolution, via gene conversion or selection, has been proposed as the process by which high sequence identity is maintained between duplicate CR regions^68,69^. Given the variation between thrips species in the apparent fidelity of concerted evolution between duplicate CR regions, this group could be a useful model system for examining these phenomena in mitochondrial genomes.

### Phylogeny of the thysanoptera

Phylogenetic inference using PCGs demonstrated significant resolving power of this data source for deep-level questions within the order Thysanoptera. Results were largely insensitive to data noise (in the form of third codon positions, or the inconsistently aligned sites masked by GBlocks) and to inference method (ML vs BI) at the levels of family interrelationships and between thripid subfamilies. Genus-level relationships within the non-monophyletic Thripinae, however, were much more variable between datasets suggesting sensitivity to data noise at these levels. This is comparable to many other insect orders in that deep-level (e.g. families/superfamily) relationships are seemingly robust to analytical decisions such as taxon selection, data inclusion/exclusion or alignment method, while shallow-level relationships show differing degrees of sensitivity^64,70–74^. The root cause of these sensitivities cannot be determined at this time, as the ‘true’ phylogeny of thrips is not yet known to compare individual analyses against. In those insect orders for which robust, independent estimates of ordinal phylogeny exist, there is inconsistency as to whether including potentially noisy regions of the mitochondrial genome (such as third codon positions) results in greater or lesser congruence between the mitochondrial topology and the nuclear and/or morphological ones. For example, Diptera^71^ and Hymenoptera^72^ trees appear to be less accurate when third codon positions are included, while topological accuracy in Isoptera is improved^74,75^, and in other orders such as Psocoptera^54^ and Lepidoptera^76^ topology is the same regardless of their inclusion or exclusion. Integration of mitochondrial genomic data with ongoing efforts to understand thrips evolution from morphological^77,78^ and nuclear genomic^79^ perspectives will help to highlight potential sources of bias in mitochondrial genomes as a data source.

The thrips phylogeny inferred using sequence data for mitochondrial PCGs for 14 species compares well with previous multi-locus phylogenies at the level of family, but is quite incompatible at the level of subfamily or genus. Buckman *et al*.^25^ analyzed five genes (including one mitochondrial gene), and in two of their three analyses (ML and BI), found the same consensus interfamily relationships as found here from the majority of datasets (i.e. Fig. 4). The position of Stenurothripidae differs in the minority result from both the present study (Thripidae + (Stenurothripidae + Aeolothripidae), found here in the ML2 and ML4 datasets (Supplementary Figs. S5 and S6), versus Stenurotrhipidae + (Thripidae + Aeolothripidae) in Buckman *et al*.’s^25^ Maximum Parsimony analysis. Despite finding the same consensus topology as here (i.e. Aeolothripidae + (Stenurothripidae + Thripidae), nodal support in Buckman *et al*.^26^ was generally low for interfamily nodes, with only their Bayesian analysis finding statistically significant support. In contrast, support for interfamily nodes in the present study are generally high (100% bootstrap support or 1.0 posterior probabilities), except for the node grouping Stenurothripidae with Thripidae. Both studies include a single representative of this family, a *Holarthrothrips* spp. in both cases, and while this family is quite small (just 6 species globally^12^), this instability in family resolution suggests that additional targeted sampling of the Stenurothripidae would be valuable in definitively resolving interfamily relationships within Thysanoptera.

In contrast, subfamily and genus-level relationships within the Thripidae are widely inconsistent across/among the present mitochondrial-genome based analyses, multi-locus analysis^25^ and morphology-based phylogeny of Thripidae^78^. Both our consensus topology (Fig. 4) and the alternative resolutions within Thripidae found in a minority of datasets differed significantly from these previous studies. The most striking difference is our consistent finding of Panchaetothripinae as the sister of the remaining Thripidae in all datasets and analyses. In contrast, both the multi-locus and morphological analyses resulted Panchaetothripinae to be nested well within Thripinae in a quite derived position within their respective trees^25,78^. Although the panchaetothripine representative used here, *Rhipiphorothrips*, was not amongst the taxa used in multi-locus analysis^25^, both it and all the taxa in the later study were included in morphology based analysis^78^ which found strong support for the monophyly of the subfamily. Mound & Morris (2007)^80^ found equivocal support for the placement of Panchaetothripinae (sister to the rest of the family in some analyses, derived within Thripidae in others), but more recent papers have been consistent in finding a derived position for the subfamily. The present study marks the first strong molecular evidence for the placement as sister to the rest of Thripidae. The sister group relationship between *Thrips* and *Frankliniella* (supported here by analyses BI-1, 2, 4 and ML-3, 4) is supported by morphology tree^78^, but was strongly rejected by the multi-locus study^25^, with the two genera consistently placed in different major clades within Thripidae. Morphological analyses suggest that the two genera are not particularly closely related, and the sister-grouping in the present study is likely due to the greatly reduced taxon sampling relative to Zhang *et al*.^78^. Considerable additional sampling is needed to resolve the relationship of these two genera which include the bulk of pest thrips species. The only consistent relationship found between the three studies is the sister-grouping of *Neohydatothrips* and *Scirtothrips* which has been suggested previously based on morphological similarities^81^, and was further supported in a recent morphological phylogeny of the *Scirtothrips* genus-group^77^. Overall, the variation in genus-level relationships found between studies that differ in taxon selection and data source, points to the difficulty in definitively resolving relationships within the Thripidae. Given the size of this family (300 genera and over 2100 species), its importance as pests, and our understanding of the evolution of feeding behaviours in thrips^78^, considerable additional thripid sampling is needed, as the use of exemplar taxa as surrogates for monophyletic higher-level groups (e.g. subfamilies or genus-groups) will be problematic.

### Genome rearrangements in thrips

The current study provides additional evidence that Thysanoptera is an insect order with heightened rates of mitochondrial genome rearrangement, consistent with Cameron’s hypothesis that haplodiploidy is a predisposing factor to genome rearrangements^1^. The four newly sequenced thrips species display three novel gene orders relative to other thrips, including the first instance of a gene order conserved over deeper evolutionary history, between representatives of the families Stenurothripidae and Thripidae (*Holarthrothrips* and *Rhipiphorothrips* respectively). Previous studies had shown that mitochondrial gene order varied between even different species of the genus^14,18^, so conserved gene orders over this scale was unexpected. The majority of genes in all thrips sequenced to date are rearranged relative to the inferred ancestral insect mitochondrial genome with only 14 of the 37 gene boundaries conserved in any of the thrips species, and the majority of the conserved gene blocks (Fig. 6) lost in some or all taxa. Indeed, of the eight conserved gene blocks identified, only block B (*atp8-atp6*) comes close to being universally conserved within thrips, being present in all species except *Neohydatothrips* (where *atp8* is inverted but not translocated). Conversely, through mapping derived gene boundaries we were able to identify several that are synapomorphic for major clades within thrips and that provide supporting evidence for the consensus topology (Fig. 4). For example, *trnW-nad1* (derived boundary #17) is synapomorphic for Stenurothripidae + Thripidae, a relationship which received equivocal support in prior molecular phylogenies of the order (Buckman *et al*., 2013), and which was sensitive to the inclusion of third codon positions in the current study. Similarly, three derived boundaries, *trnG*-*trnK* (#6), *trnK*-*cox3* (#7), and *trnY*-*nad2* (#16), were synapomorphic for the monophyly of Thripidae excluding Panchaetothripinae, another relationship which was not supported in prior molecular^25^ or morphological analyses^78^. Tubulifera is supported by seven derived gene boundaries (Fig. 5; #61 is homoplastic, 66–71 are synapomorphic), including two derived gene blocks, *nad1-cox1-trnQ-cox2* and *trnG-trnD-rrnS*, despite otherwise quite dissimilar gene arrangements (c.f. Fig. 5). Although gene rearrangements in thrips are relatively noisy (i.e., 90 unique derived gene boundaries vs 71 shared vs 24 apparently synapomorphic ones), they do provide additional evidence for clade monophyly and can complement sequence-based analyses, thus increasing our confidence in clades which are not consistently recovered between different studies or for which nodal support is weak.

As in other insect orders, the majority of rearrangements involve tRNAs (Fig. 5) adding further evidence for the selective neutrality of tRNA position within the mitochondrial genome^64^. As a consequence of the scale of tRNA positional differences between thrips mitochondrial genomes, CREx inference of the evolutionary events underlying these genome rearrangements consistently inferred multiple TDRL events encompassing duplications of major portions of the genome (Supplementary Figs. S7 and S8). This software has similarly inferred high numbers of large-scale TDRLs in other insect groups with high rates of tRNA rearrangement such as Psocoptera^54^ and Hymenoptera^7^, suggesting that it algorithmically favours TDRL events over other inferred classes of gene rearrangements. Noise from tRNA rearrangements (there are likely multiple tRNA rearrangements along each branch, analogous to the ‘multiple-hits’ problem with third-codon positions^82^), combined with the presence of duplicated tRNAs in some species and the absence of a tRNA gene from *Gynaikothrips*, made the inference of ancestral genomes for key clades within thrips computationally intractable with TreeREx (which has the same core algorithm as CREx). By limiting our ancestral genome analysis to just the major genes (PCGs and rRNAs) we were, however, able to reconstruct rearrangement of these larger, less ‘mobile’ genes. Removing tRNAs from ancestral genome reconstruction has been useful in understanding the major genome evolutionary events within Metazoa^34^, as their higher relative rates of rearrangement obscured the fundamentally conserved nature of gene arrangement across taxa as deep as superphyla. tRNA removal is similarly effective in thrips, allowing the identification of major rearrangements in the common ancestors of major clades such as Terebrantia, Stenurothripidae + Thripidae and ‘core’ Thripinae (including *Neohydatothrips*).

Direct use of gene arrangement data in phylogenetic inference using MLGO proved unsuccessful likely due of the extremely high rates of tRNA rearrangements in thrips as discussed above. The overall topology differed significantly from all the trees produced in the present study, and from previous molecular phylogenies of the order^25,78^, most notably in the non-monophyly of Terebrantia and Thripidae. Even considering gene-rearrangement data in isolation, clades that were supported by unambiguously synapomorphic rearrangements, including *Scirtothrips* + *Neohydatothrips*, Stenurothripidae + Thripidae and Thripidae excluding Panchaetothripidae, were not recovered by the MLGO analysis. Examination of novel gene boundaries (Fig. 5) clearly shows that there are large numbers of derived gene-boundaries that are either homoplastic or have been secondarily lost in one or more thrips lineages (47 of 71 derived boundaries). These, plus the high number of unique gene boundaries (90 found in just once in thrips), suggest that MLGO may be vulnerable to ‘long-branch’ type effects^83^. The two main clades that were inferred, Thripidae (except *Rhipiphorothrips*) in one and the remaining thrips species in the other, roughly correspond to the scale of total rearrangements (higher in the first clade), rather than adequately modelling phylogenetically informative versus uninformative rearrangements. Indeed, the only two thrips species to share identical gene orders (*Holarthrothrips* and *Rhipiphorothrips*) are strongly grouped as a clade, despite every other line of phylogenetic evidence suggesting that they are distantly related (morphology, multilocus and mitochondrial genome phylogenies). Properly modelling symplesiomorphic gene arrangements (as the absence of rearrangements between these species can be regarded) will likely always prove challenging given the overall low total number of characters provided by the mitochondrial genome (only 37 genes plus CR). Further exploration of MLGO and other methods of direct phylogenetic inference from gene-order data, however, will improve our understanding of how these methods work with empirical data. Further data from thrips would contribute to those explorations, especially for testing utility at finer taxonomic scales such as within families or subfamilies.

## Supplementary information

Supplementary Info.

## Data Availability

Annotated mitochondrial genome assemblies are deposited in NCBI GenBank under the following accession numbers: *F*. *vespiformis* (MN072395), *H*. *indicus* (MN072397), *R*. *cruentatus* (MN072396), *G*. *uzeli* (MK940484).
