# Supplementary material for: Rearrangement and evolution of mitochondrial genomes in Thysanoptera (Insecta)
Source: Sci Rep. 2020 Jan 20;10:695. doi: 10.1038/s41598-020-57705-4 (PMC6971079; doi:10.1038/s41598-020-57705-4)

**Supplementary Information**

**Rearrangement and evolution of mitochondrial genomes in Thysanoptera (Insecta)**

Kaomud Tyagi^1^, Rajasree Chakraborty^1^, Stephen L. Cameron^2^, Andrew D. Sweet^2^, Kailash Chandra^1^, Vikas Kumar^1*^

^1^Centre for DNA Taxonomy, Molecular Systematics Division, Zoological Survey of India, Kolkata-750053

^2^Department of Entomology, Purdue University, West Lafayette, IN 47907

*Corresponding author email id vikaszsi77@gmail.com

| Sl. No. | PCGs without G Block | | | | PCGs With Gblock excluding 3^rd^ codon position | | | |
| --- | --- | --- | --- | --- | --- | --- | --- | --- |
|  | Codon alinged | | Excluding 3^rd^ codon | | Codon alinged | | Excluding 3^rd^ codon | |
|  | Model (10) | Gene | Model | Gene | Model (9) | Gene | Model (4) | Gene |
| 1 | **GTR+I+G** | ATP6_pos1, NAD3_pos1, NAD1_pos1, NAD4L_pos1, NAD5_pos1, NAD4_pos1 |  |  | **GTR+I+G** | ATP6_pos1, NAD3_pos1, NAD1_pos1, NAD4L_pos1, NAD5_pos1, NAD4_pos1 | **K81UF+I+G** | ATP8_pos1, ATP8_pos2, ATP8_pos3, COII_pos3, ATP6_pos1, COII_pos1, COII_pos2, ATP6_pos3 |
| 2 | **GTR+I+G** | NAD4_pos2, NAD5_pos2, NAD4L_pos2, NAD3_pos2, ATP6_pos2, NAD1_pos2 |  |  | **GTR+I+G** | NAD5_pos2, NAD4_pos2, COIII_pos2, ATP6_pos2, NAD1_pos2 | **TVM+I+G** | NAD3_pos2, NAD3_pos1, NAD1_pos1, NAD1_pos3, ATP6_pos2, NAD4L_pos1, NAD3_pos3, NAD1_pos2, NAD5_pos2, NAD4_pos3, NAD5_pos3, NAD4_pos2, NAD5_pos1, NAD4_pos1 |
| 3 | **TIM+I+G** | NAD1_pos3, ATP6_pos3, COI_pos3, COII_pos3, CYTB_pos3, COIII_pos3, NAD3_pos3 |  |  | **HKY+G** | NAD6_pos3, NAD2_pos3, NAD1_pos3, ATP6_pos3 | **TIM+I+G** | COI_pos3, COI_pos2, COI_pos1, COIII_pos1, CYTB_pos3, CYTB_pos1, CYTB_pos2, COIII_pos3, COIII_pos2 |
| 4 | **TVM+I+G** | NAD2_pos1, ATP8_pos1, NAD6_pos1 |  |  | **TVM+I+G** | ATP8_pos1, ATP8_pos2, COII_pos2, NAD6_pos2, NAD3_pos2, NAD4L_pos2, NAD2_pos2 | **TVM+I+G** | NAD4L_pos3, NAD6_pos3, NAD6_pos1, NAD4L_pos2, NAD6_pos2, NAD2_pos3, NAD2_pos1, NAD2_pos2 |
| 5 | **TVM+I+G** | ATP8_pos2, NAD6_pos2, NAD2_pos2 |  |  | **HKY+G** | NAD5_pos3, NAD4_pos3, NAD4L_pos3, ATP8_pos3 |  |  |
| 6 | **HKY+G** | ATP8_pos3, NAD2_pos3, NAD6_pos3 |  |  | **GTR+I+G** | COIII_pos1, COII_pos1, COI_pos1, CYTB_pos1 |  |  |
| 7 | **GTR+I+G** | COI_pos1, COII_pos1, CYTB_pos1, COIII_pos1 |  |  | **TVM+I+G** | COI_pos2, CYTB_pos2 |  |  |
| 8 | **TVM+I+G** | COI_pos2, COIII_pos2, CYTB_pos2 |  |  | **TIM+G** | COIII_pos3, COI_pos3, CYTB_pos3, NAD3_pos3, COII_pos3 |  |  |
| 9 | **F81+G** | COII_pos2 |  |  | **K81UF+I+G** | NAD2_pos1, NAD6_pos1 |  |  |
| 10 | **HKY+G** | NAD4L_pos3, NAD4_pos3, NAD5_pos3 |  |  |  |  |  |  |

**Table S1. List of the best partition models portioned by Partition finder Analysis.**

**Table S2**. Derived characters for each taxa of Thysanoptera. Genes marked with a superscript “-” are encoded on the minority strand. Shared derived characters are labelled with codes used on figures 5 and 6.

| ***S. dorsalis* EA** | **Code** | ***S. dorsalis* SA** | **Code** | ***N. samayunkur*** | **Code** | ***T. palmi*** | **Code** | ***T. imaginis*** | **Code** | ***F. occidentalis*** | **Code** | ***F. intonsa*** | **Code** |
| --- | --- | --- | --- | --- | --- | --- | --- | --- | --- | --- | --- | --- | --- |
| **COX1-NAD3** | **1** | **COX1-NAD3** | **1** | **COX1-NAD3** | **1** | **COX1-NAD3** | **1** | **COX1-NAD3** | **1** | **COX1-NAD3** | **1** | **COX1-NAD3** | **1** |
| NAD3-L2 | 2 | NAD3-L2 | 2 | NAD3-COX2 | 31 | NAD3-COX2 | 31 | NAD3-L2 | 2 | NAD3-L2 | 2 | NAD3-L2 | 2 |
| L2-COX2 |  | L2-COX2 |  | COX2-D | 3 | **COX2-G** | **35** | L2-COX2 |  | L2-COX2 |  | L2-COX2 |  |
| COX2-D | 3 | COX2-D | 3 | D-R | 4 | **G-K** | **6** | **COX2-G** | **35** | COX2-D | 3 | COX2-D | 3 |
| D-R | 4 | D-R | 4 | R-G | 5 | **K-COX3** | **7** | **G-K** | **6** | D-R | 4 | D-R | 4 |
| R-G | 5 | R-G | 5 | **G-K** | **6** | COX3-N |  | **K-COX3** | **7** | R-G | 5 | R-G | 5 |
| **G-K** | **6** | **G-K** | **6** | **K-COX3** | **7** | N-T |  | COX3-R |  | **G-K** | **6** | **G-K** | **6** |
| **K-COX3** | **7** | **K-COX3** | **7** | COX3-N |  | T-S1 | 36 | R-T |  | **K-COX3** | **7** | **K-COX3** | **7** |
| COX3-I | 8 | COX3-I | 8 | N-E | 12 | **S1-L1** | **37** | T-N |  | COX3-T |  | COX3-I | 8 |
| **I-L1** | **9** | **I-L1** | **9** | E-I |  | L1-P^-^ | 38 | N-E | 12 | T-Q | 33 | I-T |  |
| **L1-T** | **10** | **L1-T** | **10** | I-CYTB | 32 | P^-^-I | 39 | E-S1^-^ | 44 | Q-I | 46 | T-CYTB |  |
| T-P |  | T-P |  | CYTB-Y^-^ | 15 | I-CYTB | 32 | S1^-^-P^-^ |  | I-CYTB | 32 | CYTB-Q |  |
| P-N | 11 | P-N | 11 | **Y^-^-NAD2** | **16** | CYTB-Y^-^ | 15 | P^-^-I | 39 | CYTB-P^-^ |  | Q-P^-^ | 34 |
| N-E | 12 | N-E | 12 | NAD2-W |  | **Y^-^-NAD2** | **16** | I-CYTB | 32 | **P^-^-Y^-^** | **47** | **P^-^-Y^-^** | **47** |
| **E-Q** | **13** | **E-Q** | **13** | **W-NAD1** | **17** | NAD2-W |  | CYTB-Y^-^ | 15 | **Y^-^-NAD2** | **16** | **Y^-^-NAD2** | **16** |
| Q-CYTB | 14 | Q-CYTB | 14 | NAD1-M | 18 | **W-NAD1** | **17** | **Y^-^-NAD2** | **16** | NAD2-W |  | NAD2-W |  |
| CYTB-Y^-^ | 15 | CYTB-Y | 15 | M-F |  | NAD1-M | 18 | NAD2-W |  | **W-NAD1** | **17** | **W-NAD1** | **17** |
| **Y-NAD2** | **16** | **Y-NAD2** | **16** | F-RRNS | 21 | M-A | 19 | **W-NAD1** | **17** | NAD1-M | 18 | NAD1-M | 18 |
| NAD2-W |  | NAD2-W |  | RRNS-ATP8^-^ | 22 | A-F | 20 | NAD1-M | 18 | M-A | 19 | M-A | 19 |
| **W-NAD1** | **17** | **W-NAD1** | **17** | ATP8^-^-ATP6 |  | F-RRNS | 21 | M-A | 19 | A-F | 20 | A-F^-^ | 20 |
| NAD1-M | 18 | NAD1-M | 18 | ATP6-L1 |  | RRNS-ATP8 | 22 | A-F | 20 | F-RRNS | 21 | F^-^-RRNS | 21 |
| M-A | 19 | M-A | 19 | **L1-T** | **10** | ATP8-ATP6 |  | F-RRNS | 21 | RRNS-ATP8 | 22 | RRNS-ATP8 | 22 |
| A-F | 20 | A-F^-^ | 20 | T-Q | 33 | **ATP6-Q** | **40** | RRNS-ATP8 | 22 | ATP8-ATP6 |  | ATP8-ATP6 |  |
| F-RRNS | 21 | F^-^-RRNS | 21 | Q-P^-^ | 34 | **Q-S2** | **41** | ATP8-ATP6 |  | **ATP6-N** | **48** | **ATP6-N** | **48** |
| RRNS-ATP8 | 22 | RRNS-ATP8 | 22 | P^-^-A |  | **S2-D** | **42** | **ATP6-Q** | **40** | N-E | 12 | N-E | 12 |
| ATP8-ATP6 |  | ATP8-ATP6 |  | A-NAD5^-^ |  | D-L2 |  | **Q-S2** | **41** | E-S1 | 44 | E-S1 | 44 |
| ATP6-S1 | 23 | ATP6-S1 | 23 | NAD5^-^-H^-^ |  | L2-E |  | **S2-D** | **42** | **S1-L1** | **37** | **S1-L1** | **37** |
| S1-NAD5^-^ | 24 | S1-NAD5^-^ | 24 | H^-^-NAD4^-^ |  | E-R |  | D-S1^-^ |  | **L1-NAD5^-^** | **49** | **L1-NAD5^-^** | **49** |
| NAD5^-^-H^-^ |  | NAD5^-^-H^-^ |  | NAD4^-^-NAD4L^-^ |  | R-NAD5 |  | **S1^-^-L1** | **37** | NAD5^-^-H^-^ |  | NAD5^-^-H^-^ |  |
| H^-^-NAD4^-^ |  | H^-^-NAD4^-^ |  | NAD4L^-^-V | 30 | NAD5-H |  | L1-E |  | H^-^-NAD4^-^ |  | H^-^-NAD4^-^ |  |
| NAD4^-^-NAD4L^-^ |  | NAD4^-^-NAD4L^-^ |  | V-NAD6 |  | H-NAD4 |  | E-NAD5^-^ | 45 | NAD4^-^-NAD4L^-^ |  | NAD4^-^-NAD4L^-^ |  |
| NAD4L^-^-C | 25 | NAD4L^-^-V | 30 | NAD6-RRNL |  | NAD4-NAD4L |  | NAD5^-^-H^-^ |  | NAD4L^-^-C | 25 | NAD4L^-^-C | 25 |
| C-NAD6 | 26 | V-RRNL |  | RRNL-S1 |  | NAD4L-C | 25 | H^-^-NAD4^-^ |  | C-NAD6 | 26 | C-NAD6 | 26 |
| NAD6-V | 27 | RRNL-S2 | 28 | S1-C |  | C-NAD6 | 26 | NAD4^-^-NAD4L^-^ |  | NAD6-V | 27 | NAD6-V | 27 |
| V-RRNL |  | S2-C |  | C-L2 |  | NAD6-V | 27 | NAD4L^-^-C | 25 | V-RRNL |  | V-RRNL |  |
| RRNL-S2 | 28 | C-NAD6 | 26 | L2-S2 |  | V-RRNL |  | C-NAD6 | 26 | RRNL-S2 | 28 | RRNL-S2 | 28 |
| S2-COX1 | 29 | NAD6-COX1 |  | S2-COX1 | 29 | RRNL-COX1 | 43 | NAD6-V | 27 | S2-COX1 | 29 | S2-COX1 | 29 |
|  |  |  |  |  |  |  |  | V-RRNL |  |  |  |  |  |
|  |  |  |  |  |  |  |  | RRNL-COX1 | 43 |  |  |  |  |
| ***D. minowai*** | **Code** | ***A. obscurus*** | **Code** | ***R. cruentatus*/ *H. indicus*** | **Code** | ***F. vespiformis*** | **Code** | ***H. aculeatus*** | **Code** | ***G. uzeli*** | **Code** |  |  |
| COX1-L2 |  | COX1-L2 |  | COX1-L2 |  | COX1-S1 |  | **COX1-Q** | **66** | **COX1-Q** | **66** |  |  |
| L2-COX2 |  | L2-COX2 |  | L2-COX2 |  | S1-K |  | **Q-COX2** | **67** | **Q-COX2** | **67** |  |  |
| COX2-D | 3 | COX2-D | 3 | COX2-L1 | 55 | K-NAD1 |  | COX2-A |  | COX2-ATP8 |  |  |  |
| D-R | 4 | D-R | 4 | L1-COX3 | 56 | NAD1-L2 |  | A-L1 |  | ATP8-ATP6 |  |  |  |
| R-G | 5 | R-G | 5 | COX3-NAD3 | 50 | L2-COX2 |  | L1-ATP8 |  | ATP6-COX3 |  |  |  |
| **G-K** | **6** | **G-K** | **6** | NAD3-R | 57 | COX2-L1 | 55 | ATP8-ATP6 |  | COX3-NAD3 | 50 |  |  |
| **K-COX3** | **7** | **K-COX3** | **7** | R-N |  | L1-COX3 | 56 | ATP6-F |  | NAD3-N | 51 |  |  |
| COX3-NAD3 | 50 | COX3-NAD3 | 50 | N-E | 12 | COX3-NAD3 | 50 | F-RRNL |  | N-S1 |  |  |  |
| NAD3-N | 51 | NAD3-N | 51 | E-Q | 13 | NAD3-N | 51 | RRNL-C^-^ |  | S1-E |  |  |  |
| N-E | 12 | N-E | 12 | Q-I | 46 | N-RRNS |  | C^-^-NAD2 |  | E-NAD5^-^ | 45 |  |  |
| E-Q | 13 | E-Q | 13 | I-CYTB | 32 | RRNS-W |  | NAD2-G |  | NAD5^-^-L1^-^ |  |  |  |
| Q-I | 29 | Q-I | 29 | CYTB-Y^-^ | 15 | W-R |  | **G-D** | **68** | L1^-^-F |  |  |  |
| I-CYTB | 32 | I-CYTB | 32 | Y^-^-G | 58 | R-E |  | D-RRNS | 61 | F-H^-^ |  |  |  |
| CYTB-Y^-^ | 15 | CYTB-Y^-^ | 15 | G-NAD2 | 59 | E-Q | 13 | RRNS-T^-^ | 54 | H^-^-S2^-^ |  |  |  |
| **Y^-^-NAD2** | **16** | **Y^-^-NAD2** | **16** | NAD2-W |  | Q-I | 46 | T^-^-NAD4L |  | S2^-^-C^-^ |  |  |  |
| NAD2-W |  | NAD2-W |  | **W-NAD1** | **17** | I-CYTB | 32 | NAD4L-NAD4 |  | C^-^-NAD4L^-^ | 25 |  |  |
| **W-NAD1** | **17** | **W-NAD1** | **17** | NAD1-D | 60 | CYTB-Y^-^ | 15 | NAD4-S2 |  | NAD4L^-^-NAD4^-^ |  |  |  |
| NAD1-M | 18 | NAD1-A |  | D-RRNS | 61 | Y^-^-G | 58 | S2-P |  | NAD4^-^-T |  |  |  |
| M-A | 19 | A-F | 20 | RRNS-K | 62 | G-NAD2 | 59 | P-I | 39 | T-NAD6 |  |  |  |
| A-F | 20 | F-RRNS | 21 | K-ATP8 | 63 | NAD2-ATP8 |  | I-W |  | NAD6-CYTB |  |  |  |
| F-RRNS | 21 | RRNS-T | 54 | ATP8-ATP6 |  | ATP8-ATP6 |  | W-K |  | **CYTB-R** | **70** |  |  |
| RRNS-ATP8 | 22 | T-M |  | ATP6-S1 | 23 | ATP6-NAD5^-^ |  | **K-M** | **69** | R^-^-A^-^ |  |  |  |
| ATP8-ATP6 |  | M-ATP8 |  | S1-NAD5^-^ | 24 | NAD5^-^-H^-^ |  | M-V |  | A^-^-Y^-^ |  |  |  |
| ATP6-C |  | ATP8-ATP6 |  | NAD5^-^-H^-^ |  | H^-^-NAD4^-^ |  | V-M |  | Y^-^-RRNS^-^ |  |  |  |
| C-T |  | ATP6-S1 | 23 | H^-^-NAD4^-^ |  | NAD4^-^-NAD4L^-^ |  | M-L2 |  | RRNS^-^-D^-^ | 61 |  |  |
| T-S1 | 36 | S1-NAD5^-^ | 24 | NAD4^-^-NAD4L^-^ |  | NAD4L^-^-T |  | L2-R |  | **D^-^-G^-^** | **68** |  |  |
| S1-NAD5^-^ | 24 | NAD5^-^-H^-^ |  | NAD4L^-^-T |  | T-P^-^ |  | **R-CYTB^-^** | **70** | G^-^-L2^-^ |  |  |  |
| NAD5^-^-H^-^ |  | H^-^-NAD4^-^ |  | T-P^-^ |  | P^-^-NAD6 |  | CYTB^-^-H |  | L2^-^-W^-^ |  |  |  |
| H^-^-NAD4^-^ |  | NAD4^-^-NAD4L^-^ | | P^-^-NAD6 |  | NAD6-C^-^ | 26 | H-NAD5 |  | W^-^-M^-^ |  |  |  |
| NAD4^-^-NAD4L^-^ |  | **NAD4L^-^-L1** | **52** | NAD6-C^-^ | 26 | C^-^-M | 64 | NAD5-E^-^ | 45 | **M^-^-K^-^** | **69** |  |  |
| **NAD4L^-^-L1** | **52** | L1-C |  | C^-^-M | 64 | M-A | 19 | E-S1 |  | K^-^-RRNL^-^ |  |  |  |
| L1-P^-^ | 38 | C-NAD6 | 26 | M-A | 19 | A-F | 20 | S1-N^-^ |  | RRNL^-^-P^-^ |  |  |  |
| **P^-^-NAD6** | **53** | **NAD6-P^-^** | **53** | A-F | 20 | F-V | 65 | N^-^-NAD3^-^ | 51 | P^-^-NAD2^-^ |  |  |  |
| NAD6-V | 27 | P^-^-V |  | F-V | 65 | V-RRNL |  | NAD3^-^-COX3^-^ | 50 | NAD2^-^-V^-^ |  |  |  |
| V-RRNL |  | V-RRNL |  | V-RRNL |  | RRNL-S2 | 28 | COX3^-^-NAD6^-^ |  | V^-^-NAD1 |  |  |  |
| RRNL-S2 | 28 | RRNL-S2 | 28 | RRNL-S2 | 28 | S2-D | 42 | NAD6^-^-Y^-^ |  | **NAD1-COX1** | **72** |  |  |
| S2-COX1 | 29 | S2-COX1 | 29 | S2-COX1 | 29 | D-COX1 |  | Y^-^-NAD1 |  |  |  |  |  |
|  |  |  |  |  |  |  |  | **NAD1-COX1** | **72** |  |  |  |  |

**Table S3. CREX analysis imput file**

>AB

cox1 L2 cox2 K D atp8 atp6 cox3 G nad3 A R N S1 E -F -nad5 -H -nad4 -nad4L T -P nad6 cytb S2 -nad1 -L1 -rrnL -V -rrnS Q M nad2 W -C -Y

>NS

cox1 nad3 cox2 D R G K cox3 N E cytb -Y nad2 W nad1 M F rrnS -atp8 atp6 L1 T Q -P A -nad5 -H -nad4 -nad4L V nad6 rrnL S1 C L2 S2

>TI

cox1 nad3 L2 cox2 G K cox3 R T N E -P cytb -Y nad2 W nad1 M A F rrnS atp8 atp6 Q S2 D -S1 L1 E -nad5 -H -nad4 -nad4L C nad6 V rrnL

>FI

cox1 nad3 L2 cox2 D R G K cox3 T cytb Q -P -Y nad2 W nad1 M A F rrnS atp8 atp6 N E S1 L1 -nad5 -H -nad4 -nad4L C nad6 V rrnL S2

>FO

cox1 nad3 L2 cox2 D R G K cox3 T Q cytb -P -Y nad2 W nad1 M A F rrnS atp8 atp6 N E S1 L1 -nad5 -H -nad4 -nad4L C nad6 V rrnL S2

>SDEA

cox1 nad3 L2 cox2 D R G K cox3 L1 T P N E Q cytb -Y nad2 W nad1 M A F rrnS atp8 atp6 S1 -nad5 -H -nad4 -nad4L C nad6 V rrnL S2

>SDSA

cox1 nad3 L2 cox2 D R G K cox3 L1 T P N E Q cytb Y nad2 W nad1 M A -F rrnS atp8 atp6 S1 -nad5 -H -nad4 -nad4L V rrnL S2 C nad6

>TP

cox1 nad3 cox2 G K cox3 N T S1 L1 -P cytb -Y nad2 W nad1 M A F rrnS atp8 atp6 Q S2 D L2 E R -nad5 -H -nad4 -nad4L C nad6 V rrnL

>AO

cox1 L2 cox2 D R G K cox3 nad3 N E Q cytb -Y nad2 W nad1 A F rrnS T M atp8 atp6 S1 -nad5 -H -nad4 -nad4L L1 C nad6 -P V rrnL S2

>GU

cox1 Q cox2 atp8 atp6 cox3 nad3 N S1 E -nad5 -L1 F -H -S2 -C -nad4L -nad4 T nad6 cytb -R -A -Y -rrnS -D -G -L2 -W -M -K -rrnL -P -nad2 -V nad1

>HI

cox1 L2 cox2 L1 cox3 nad3 R N E Q cytb -Y G nad2 W nad1 D rrnS K atp8 atp6 S1 -nad5 -H -nad4 -nad4L T -P nad6 -C M A F V rrnL S2

>FV

cox1 S1 K nad1 L2 cox2 L1 cox3 nad3 N rrnS W R E Q cytb -Y G nad2 atp8 atp6 -nad5 -H -nad4 -nad4L T -P nad6 -C M A F V rrnL S2 D

>RC

cox1 L2 cox2 L1 cox3 nad3 R N E Q cytb -Y G nad2 W nad1 D rrnS K atp8 atp6 S1 -nad5 -H -nad4 -nad4L T -P nad6 -C M A F V rrnL S2

>DM

cox1 L2 cox2 D R G K cox3 nad3 N E Q cytb -Y nad2 W nad1 M A F rrnS atp8 atp6 C T S1 -nad5 -H -nad4 -nad4L L1 -P nad6 V rrnL S2

>HA

cox1 Q cox2 A L1 atp8 atp6 F rrnL -C nad2 G D rrnS -T nad4L nad4 S2 P W K V M L2 R -cytb H nad5 -E S1 -N -nad3 -cox3 -nad6 -Y nad1

**Table S4. TreeREx analysis imput file**

**(Including Protein coding genes, Ribosomal genes and one copy of Control region)**

>AB

cox1 cox2 atp8 atp6 cox3 nad3 -nad5 -nad4 -nad4L nad6 cytb -nad1 -rrnL -rrnS CR nad2

>NS

cox1 nad3 cox2 cox3 cytb nad2 nad1 rrnS -atp8 atp6 -nad5 -nad4 -nad4L CR nad6 rrnL

>TI

cox1 nad3 cox2 cox3 cytb nad2 nad1 rrnS atp8 atp6 CR -nad5 -nad4 -nad4L nad6 rrnL

>FI

cox1 nad3 cox2 cox3 cytb nad2 nad1 rrnS atp8 atp6 CR -nad5 -nad4 -nad4L nad6 rrnL

>FO

cox1 nad3 cox2 cox3 cytb nad2 nad1 rrnS atp8 atp6 CR -nad5 -nad4 -nad4L nad6 rrnL

>SDEA

cox1 nad3 cox2 cox3 cytb nad2 nad1 rrnS atp8 atp6 CR -nad5 -nad4 -nad4L nad6 rrnL

>TP

cox1 nad3 cox2 cox3 cytb nad2 nad1 rrnS atp8 atp6 CR -nad5 -nad4 -nad4L nad6 rrnL

>AO

cox1 cox2 cox3 nad3 cytb nad2 nad1 rrnS atp8 atp6 CR -nad5 -nad4 -nad4L nad6 rrnL

>HI

cox1 cox2 cox3 nad3 cytb nad2 nad1 rrnS atp8 atp6 CR -nad5 -nad4 -nad4L nad6 rrnL

>FV

cox1 nad1 cox2 cox3 nad3 rrnS CR cytb nad2 atp8 atp6 -nad5 -nad4 -nad4L nad6 rrnL

>RC

cox1 cox2 cox3 nad3 cytb nad2 nad1 rrnS atp8 atp6 CR -nad5 -nad4 -nad4L nad6 rrnL

>DM

cox1 cox2 cox3 nad3 cytb nad2 nad1 rrnS atp8 atp6 CR -nad5 -nad4 -nad4L nad6 rrnL

>GU

cox1 cox2 atp8 atp6 cox3 nad3 -nad5 -nad4 -nad4L nad6 cytb -rrnS -rrnL CR -nad2 nad1

>HA

cox1 cox2 atp8 atp6 CR rrnL nad2 rrnS nad4L nad4 -cytb nad5 -nad3 -cox3 -nad6 nad1

**Table S5. MLGO analysis imput file**

>AB

cox1 L2 cox2 K D atp8 atp6 cox3 G nad3 A R N S1 E -F -nad5 -H -nad4 -nad4L T -P nad6 cytb S2 -nad1 -L1 -rrnL -V -rrnS I Q M nad2 W -C -Y

>NS

cox1 nad3 cox2 D R G K cox3 N E I cytb -Y nad2 W nad1 M F rrnS -atp8 atp6 L1 T Q -P A -nad5 -H -nad4 -nad4L V nad6 rrnL S1 C L2 S2

>TI

cox1 nad3 L2 cox2 G K cox3 R T N E S1 -P I cytb -Y nad2 W nad1 M A F rrnS atp8 atp6 Q S2 D -S1 L1 E -nad5 -H -nad4 -nad4L C nad6 V rrnL

>FI

cox1 nad3 L2 cox2 D R G K cox3 I T cytb Q -P -Y nad2 W nad1 M A F rrnS atp8 atp6 N E S1 L1 -nad5 -H -nad4 -nad4L C nad6 V rrnL S2

>FO

cox1 nad3 L2 cox2 D R G K cox3 T Q I cytb -P -Y nad2 W nad1 M A F rrnS atp8 atp6 N E S1 L1 -nad5 -H -nad4 -nad4L C nad6 V rrnL S2

>SDEA

cox1 nad3 L2 cox2 D R G K cox3 I L1 T P N E Q cytb -Y nad2 W nad1 M A F rrnS atp8 atp6 S1 -nad5 -H -nad4 -nad4L C nad6 V rrnL S2

>SDSA

cox1 nad3 L2 cox2 D R G K cox3 I L1 T P N E Q cytb Y nad2 W nad1 M A -F rrnS atp8 atp6 S1 -nad5 -H -nad4 -nad4L V rrnL S2 C nad6

>TP

cox1 nad3 cox2 G K cox3 N T S1 L1 -P I cytb -Y nad2 W nad1 M A F rrnS atp8 atp6 Q S2 D L2 E R -nad5 -H -nad4 -nad4L C nad6 V rrnL

>AO

cox1 L2 cox2 D R G K cox3 nad3 N E Q I cytb -Y nad2 W nad1 A F rrnS T M atp8 atp6 S1 -nad5 -H -nad4 -nad4L L1 C nad6 -P V rrnL S2

>GU

cox1 Q cox2 atp8 atp6 cox3 nad3 N S1 E -nad5 -L1 F -H -S2 -C -nad4l –nad4 T nad6 cytb -R -A -Y -rrnS -D -G -L2 -W -M -K -rrnL CR -P -nad2 -V nad1

>HI

cox1 L2 cox2 L1 cox3 nad3 R N E Q I cytb -Y G nad2 W nad1 D rrnS K atp8 atp6 S1 -nad5 -H -nad4 -nad4L T -P nad6 -C M A F V rrnL S2

>FV

cox1 S1 K nad1 L2 cox2 L1 cox3 nad3 N rrnS W R E Q I cytb -Y G nad2 atp8 atp6 -nad5 -H -nad4 -nad4L T -P nad6 -C M A F V rrnL S2 D

>RC

cox1 L2 cox2 L1 cox3 nad3 R N E Q I cytb -Y G nad2 W nad1 D rrnS K atp8 atp6 S1 -nad5 -H -nad4 -nad4L T -P nad6 -C M A F V rrnL S2

>DM

cox1 L2 cox2 D R G K cox3 nad3 N E Q I cytb -Y nad2 W nad1 M A F rrnS atp8 atp6 C T S1 -nad5 -H -nad4 -nad4L L1 -P nad6 V rrnL S2

>HA

cox1 Q cox2 A L1 atp8 atp6 F rrnL -C nad2 G D rrnS -T nad4L nad4 S2 P I W K M V M L2 R -cytb H nad5 -E S1 -N -nad3 -cox3 -nad6 -Y nad1

**Table S6. The mitochondrial genes of *F*. *vespiformis*, *H*. *indicus*, *R*. *cruentatus*, *G*. *uzeli*.** The PCGs and rRNA genes are represented by standard nomenclature, tRNAs are represented as *trn* followed by the IUPAC-IUB single letter amino acid codes. Genes on minority strand represented by asterisk. IGN represents (+) values as intergenic nucleotides and (-) values as overlapping regions. CR represents the control region.

| ***F. vespiformis*** | | | | | | ***Holarthrothrips indicus*** | | | | | |
| --- | --- | --- | --- | --- | --- | --- | --- | --- | --- | --- | --- |
| **Gene** | **Location** | **Size (bp)** | **Start Codon** | **Stop Codon** | **IGN** | **Gene** | **Location** | **Size (bp)** | **Start Codon** | **Stop Codon** | **IGN** |
| *cox1* | 16-1557 | 1542 | TTG | TAA | -2 | *cox1* | 10-1545 | 1536 | TTG | TAA | 5 |
| *trnS1* | 1556-1615 | 60 | - | - | 39 | *trnL2* | 1551-1617 | 67 | - | - | 34 |
| *trnK* | 1655-1718 | 64 | - | - | 66 | *cox2* | 1652-2341 | 690 | ATT | TAA | 10 |
| *nad1* | 1785-2717 | 933 | ATA | TAA | -32 | *trnL1* | 2352-2416 | 65 | - | - | 1 |
| *trnL2* | 2686-2750 | 65 | - | - | 0 | *cox3* | 2418-3200 | 783 | ATA | TAA | 26 |
| CR2 | 2751-3057 | 307 | - | - | 0 | *nad3* | 3227-3580 | 354 | ATA | TAA | -5 |
| *cox2* | 3058-3765 | 708 | ATG | TAA | 24 | *trnR* | 3576-3645 | 70 | - | - | -2 |
| *trnL1* | 3790-3855 | 66 |  |  | 53 | *trnN* | 3644-3712 | 69 | - | - | -3 |
| *cox3* | 3909-4691 | 783 | ATA | TAA | -1 | *trnE* | 3710-3773 | 64 | - | - | 58 |
| *nad3* | 4691-5044 | 354 | ATG | TAA | 0 | *trnQ* | 3832-3899 | 68 | - | - | -3 |
| *trnN* | 5045-5113 | 69 | - | - | 5 | *trnI* | 3897-3967 | 71 | - | - | 1 |
| *rrnS* | 5119-5179 | 61 | - | - | 154 | *cytb* | 3969-5096 | 1128 | ATA | TAA | -3 |
| *trnW* | 5334-5399 | 66 | - | - | 2 | *trnY** | 5094-5159 | 66 | - | - | 9 |
| *trnR* | 5402-5466 | 65 | - | - | 0 | *trnG* | 5169-5233 | 65 | - | - | 1 |
| CR1 | 5467-7000 | 1534 | - | - | 0 | *nad2* | 5235-6290 | 1056 | ATT | TAG | -1 |
| *trnE* | 7001-7066 | 66 | - | - | 2 | *trnW* | 6290-6354 | 65 | - | - | 1 |
| *trnQ* | 7069-7136 | 68 | - | - | 3 | *nad1* | 6356-7285 | 930 | ATT | TAA | 0 |
| *trnI* | 7140-7203 | 64 | - | - | 7 | *trnD* | 7286-7349 | 64 | - | - | 6 |
| *cytb* | 7211-8323 | 1113 | ATT | TAA | -3 | *rrnS* | 7356-8107 | 752 | - | - | -2 |
| *trnY** | 8321-8389 | 69 | - | - | 1 | *trnK* | 8106-8171 | 66 | - | - | 13 |
| *trnG* | 8391-8456 | 66 | - | - | 25 | *atp8* | 8185-8349 | 165 | ATT | TAA | -10 |
| *nad2* | 8482-9468 | 987 | ATA | TAA | 27 | *atp6* | 8340-9026 | 687 | ATG | TAA | 2 |
| *atp8* | 9496-9660 | 165 | ATA | TAG | -10 | *trnS1* | 9029-9093 | 65 | - | - | 0 |
| *atp6* | 9651-10337 | 687 | ATG | TAA | 180 | CR | 9094-9586 | 493 | - | - | 0 |
| *nad5** | 10518-12236 | 1719 | ATT | TAA | 18 | *nad5** | 9587-11290 | 1704 | ATT | TAA | 30 |
| *trnH** | 12255-12319 | 65 | - | - | -1 | *trnH** | 11321-11386 | 66 | - | - | 0 |
| *nad4** | 12319-13641 | 1328 | ATG | TAA | -7 | *nad4** | 11387-12715 | 1329 | ATG | TAA | -1 |
| *nad4l** | 13635-13922 | 288 | ATG | TAG | 2 | *nad4l** | 12715-13014 | 300 | ATG | TAA | 2 |
| *trnT* | 13925-13989 | 65 | - | - | 0 | *trnT* | 13017-13080 | 64 | - | - | 1 |
| *trnP** | 13990-14056 | 67 | - | - | 14 | *trnP** | 13082-13148 | 67 | - | - | 3 |
| *nad6* | 14071-14547 | 477 | ATA | TAA | -1 | *nad6* | 13152-13658 | 507 | ATT | TAA | -2 |
| *trnC** | 14547-14610 | 64 | - | - | -7 | *trnC** | 13657-13721 | 65 | - | - | 1 |
| *trnM* | 14604-14672 | 69 | - | - | 2 | *trnM* | 13723-13788 | 66 | - | - | 0 |
| *trnA* | 14675-14738 | 64 | - | - | 3 | *trnA* | 13789-13860 | 72 | - | - | -1 |
| *trnF* | 14742-14812 | 71 | - | - | 1 | *trnF* | 13860-13927 | 68 | - | - | 1 |
| *trnV* | 14814-14877 | 64 | - | - | 199 | *trnV* | 13929-13999 | 71 | - | - | 155 |
| *rrnL* | 15077-16086 | 1010 | - | - | 14 | *rrnL* | 14155-15161 | 1007 | - | - | 19 |
| *trnS2* | 16101-16168 | 68 | - | - | 1 | *trnS2* | 15181-15243 | 71 | - | - |  |
| *trnD* | 16170-16220 | 51 | - | - |  | *trnS2* | 1-8 |  |  |  |  |
| ***Rhipiphorothrips cruentatus*** | | | | | | ***Gynaikothrips uzeli*** | | | | | |
| **Gene** | **Location** | **Size (bp)** | **Start Codon** | **Stop Codon** | **IGN** | **Gene** | **Location** | **Size (bp)** | **Start Codon** | **Stop Codon** | **IGN** |
| *nad5** | 1-1519 | 1519 | ATA | TAG | 72 | *nad1* | 89-1003 | 915 | ATT | TAA | 37 |
| *trnH** | 1592-1654 | 63 | - | - | 6 | *cox1* | 1041-2591 | 1551 | ATT | TAA | -59 |
| *nad4** | 1661-2983 | 1323 | ATG | TAA | -7 | *trnQ* | 2533-2600 | 68 | - | - | 43 |
| *nad4l** | 2977-3267 | 291 | ATG | TAA | 1 | *cox2* | 2644-3228 | 585 | ATT | TAA | -76 |
| *trnT* | 3269-3330 | 62 | - | - | 1 | *atp8* | 3153-3332 | 180 | ATT | TAA | -4 |
| *trnP** | 3332-3397 | 66 | - | - | -22 | *atp6* | 3329-4000 | 672 | ATT | TAA | 20 |
| *nad6* | 3376-3882 | 507 | ATT | TAA | -2 | *cox3* | 4021-4797 | 777 | ATT | TAA | -29 |
| *trnC** | 3881-3943 | 63 | - | - | -2 | *nad3* | 4769-5143 | 375 | ATT | TAA | -4 |
| *trnM* | 3942-4002 | 61 | - | - | 0 | *trnN* | 5140-5203 | 64 | - | - | -2 |
| *trnA* | 4003-4066 | 64 | - | - | -1 | *trnS1* | 5202-5259 | 58 | - | - | 3 |
| *trnF* | 4066-4126 | 61 | - | - | -1 | *trnE* | 5263-5321 | 59 | - | - | -9 |
| *trnV* | 4126-4179 | 54 | - | - | -59 | *nad5** | 5313-6989 | 1677 | ATT | TAA | 1 |
| *rrnL* | 4121-5242 | 1122 | - | - | -25 | *trnL1** | 6991-7053 | 63 | - | - | -15 |
| *trnS2* | 5218-5284 | 67 | - | - | -8 | *trnF* | 7039-7105 | 67 | - | - | 4 |
| *cox1* | 5277-6818 | 1542 | ATT | TAA | 2 | *trnH** | 7110-7170 | 61 | - | - | 11 |
| *trnL2* | 6821-6884 | 64 | - | - | 1 | *trnS2** | 7182-7246 | 65 | - | - | 8 |
| *cox2* | 6886-7567 | 682 | ATA | T(AA) | 19 | *trnC** | 7255-7319 | 65 | - | - | 2 |
| *trnL1* | 7587-7648 | 62 | - | - | 1 | *nad4** | 7322-8632 | 1311 | ATG | TAA | -7 |
| *cox3* | 7650-8441 | 792 | ATA | TAA | 3 | *nad4l** | 8626-8886 | 261 | ATG | TAA | -2 |
| *nad3* | 8445-8798 | 354 | ATT | TAA | -2 | *trnT* | 8885-8947 | 63 | - | - | 7 |
| *trnR* | 8797-8863 | 67 | - | - | -7 | *nad6* | 8955-9431 | 477 | ATG | TAA | 39 |
| *trnN* | 8857-8923 | 67 | - | - | -3 | *cytb* | 9471-10610 | 1140 | ATT | TAA | -20 |
| *trnE* | 8921-8975 | 55 | - | - | 2 | *trnR** | 10591-10658 | 68 | - | - | 1 |
| *trnQ* | 8978-9045 | 68 | - | - | -4 | *trnA** | 10660-10722 | 63 | - | - | 4 |
| *trnI* | 9042-9108 | 67 | - | - | 1 | *trnY** | 10727-10790 | 64 | - | - | -4 |
| *cytb* | 9110-10222 | 1113 | ATA | TAA | -20 | *rrnS** | 10787-11511 | 725 | - | - | -1 |
| *trnY** | 10203-10270 | 68 | - | - | -2 | *trnD** | 11511-11576 | 66 | - | - | 5 |
| *trnG* | 10269-10330 | 62 | - | - | 37 | *trnG*** | 11582-11642 | 61 | - | - | 1 |
| *nad2* | 10368-11312 | 945 | ATA | TAA | 13 | *trnL2* | 11644-11709 | 66 | - | - | 0 |
| *trnW* | 11326-11391 | 66 | - | - | 1 | *trnW** | 11710-11775 | 66 | - | - | 0 |
| *nad1* | 11393-12316 | 924 | ATT | TAA | -2 | *trnM** | 11776-11839 | 64 | - | - | 9 |
| *trnD* | 12315-12376 | 62 | - | - | 0 | *trnK** | 11849-11922 | 74 | - | - | 0 |
| *rrnS* | 12377-13050 | 674 | - | - | -3 | *rrnL** | 11923-12598 | 676 | - | - | 1 |
| *trnK* | 13048-13109 | 62 | - | - | 1 | CR | 12600-13100 | 501 | - | - | 0 |
| *atp8* | 13111-13284 | 174 | ATT | TAA | -10 | *trnP** | 13101-13167 | 67 | - | - | 149 |
| *atp6* | 13275-13958 | 684 | ATG | TAA | -2 | *nad2** | 13317-13856 | 540 | ATG | TAA | 24 |
| *trnS1* | 13957-14018 | 62 | - | - | 0 | *trnV** | 13881-13943 | 63 | - | - | 146 |
| CR | 14019-15003 | 985 | - | - | 0 |  |  |  |  |  |  |
| *nad5** | 15004-15143 | 140 | - | - |  |  |  |  |  |  |  |

**Table S7. Nucleotide composition and skew in Thysanoptera mitochondrial genomes.**

| **Species** | **Size (bp)** | **A%** | **G%** | **T%** | **C%** | **GC%** | **AT%** | **AT skew** | **GC skew** |
| --- | --- | --- | --- | --- | --- | --- | --- | --- | --- |
| **Whole mitochondrial genome** | | | | | | | | | |
| *F.vespiformis* | 16,224 | 37.81 | 11.92 | 37.54 | 12.73 | 24.65 | 75.35 | -0.004 | 0.033 |
| *G.uzeli* | 14,002 | 43.14 | 8.82 | 39.00 | 9.03 | 17.85 | 82.15 | -0.050 | 0.012 |
| *R.cruentatus* | 15,143 | 41.37 | 9.16 | 35.20 | 14.26 | 23.42 | 76.58 | -0.081 | 0.218 |
| *H.indicus* | 15,243 | 39.27 | 11.22 | 35.62 | 13.88 | 25.11 | 74.89 | -0.049 | 0.106 |
| *D.minowai* | 14,631 | 40.93 | 9.84 | 37.60 | 11.63 | 21.47 | 78.53 | -0.042 | 0.083 |
| *N.samayunkur* | 15,295 | 40.25 | 10.98 | 37.17 | 11.60 | 22.58 | 77.42 | -0.040 | 0.027 |
| *H.aculeatus* | 14,616 | 38.47 | 9.35 | 43.53 | 8.64 | 17.99 | 82.01 | 0.062 | -0.040 |
| *T. palmi* | 15,333 | 42.71 | 10.14 | 35.58 | 11.58 | 21.72 | 78.28 | -0.091 | 0.066 |
| *T. imaginis* | 15,407 | 43.85 | 10.47 | 32.72 | 12.96 | 23.43 | 76.57 | -0.145 | 0.106 |
| *F. intonsa* | 15,215 | 41.24 | 11.06 | 34.68 | 13.01 | 24.07 | 75.93 | -0.086 | 0.081 |
| *F. occidentalis* | 14,889 | 40.98 | 11.35 | 36.62 | 11.06 | 22.41 | 77.59 | -0.056 | -0.013 |
| *S. dorsalis* EA1 | 15,343 | 39.12 | 11.61 | 36.62 | 12.64 | 24.26 | 75.74 | -0.033 | 0.042 |
| *S. dorsalis* SA1 | 15,204 | 39.83 | 11.18 | 37.56 | 11.42 | 22.6 | 77.4 | -0.029 | 0.011 |
| *A. obscurus* | 14,890 | 38.38 | 11.27 | 39.75 | 10.6 | 21.87 | 78.13 | 0.018 | -0.031 |
| **Protein coding gene** | | | | | | | | | |
| *F.vespiformis* | 11,079 | 37.15 | 12.02 | 37.42 | 13.40 | 25.43 | 74.57 | -0.004 | -0.054 |
| *G.uzeli* | 10,566 | 42.69 | 9.20 | 38.22 | 9.89 | 19.09 | 80.91 | 0.055 | -0.036 |
| *R.cruentatus* | 10,978 | 40.56 | 9.05 | 35.38 | 15.00 | 24.06 | 75.94 | 0.068 | -0.247 |
| *H.indicus* | 11,169 | 38.40 | 11.45 | 35.36 | 14.79 | 26.24 | 73.76 | 0.041 | -0.127 |
| *D.minowai* | 10,928 | 39.87 | 9.79 | 37.98 | 12.36 | 22.15 | 77.85 | 0.024 | -0.116 |
| *N.samayunkur* | 10,982 | 39.59 | 10.79 | 37.56 | 12.06 | 22.85 | 77.15 | 0.026 | -0.055 |
| *H.aculeatus* | 10,781 | 36.83 | 9.90 | 43.86 | 9.41 | 19.30 | 80.70 | -0.087 | 0.025 |
| *T. palmi* | 11,032 | 41.64 | 10.52 | 35.32 | 12.52 | 23.04 | 76.96 | 0.08 | -0.09 |
| *T. imaginis* | 10,922 | 42.75 | 10.15 | 32.89 | 14.21 | 24.36 | 75.64 | 0.13 | -0.17 |
| *F. intonsa* | 11,009 | 39.95 | 11.39 | 34.58 | 14.08 | 25.47 | 74.53 | 0.07 | -0.11 |
| *F. occidentalis* | 10,852 | 39.82 | 11.62 | 36.72 | 11.84 | 23.46 | 76.54 | 0.04 | -0.01 |
| *S. dorsalis* EA1 | 10,954 | 38.06 | 11.92 | 36.53 | 13.48 | 25.41 | 74.59 | 0.02 | -0.06 |
| *S. dorsalis* SA1 | 10,973 | 38.94 | 11.36 | 37.67 | 12.03 | 23.38 | 76.62 | 0.02 | -0.03 |
| *A. obscurus* | 11,167 | 37.36 | 11.46 | 39.93 | 11.25 | 22.71 | 77.29 | -0.03 | 0.01 |
| **Transfer RNA** | | | | | | | | | |
| *F.vespiformis* | 1,446 | 39.90 | 11.89 | 37.34 | 10.86 | 22.75 | 77.25 | 0.03 | 0.05 |
| *G.uzeli* | 1,355 | 44.65 | 6.64 | 42.07 | 6.64 | 13.28 | 86.72 | 0.03 | 0.00 |
| *R.cruentatus* | 1,430 | 41.89 | 9.65 | 37.13 | 11.33 | 20.98 | 79.02 | 0.06 | -0.08 |
| *H.indicus* | 1,483 | 41.00 | 10.72 | 37.15 | 11.13 | 21.85 | 78.15 | 0.05 | -0.02 |
| *D.minowai* | 1,428 | 42.58 | 10.15 | 37.32 | 9.94 | 20.10 | 79.90 | 0.07 | 0.01 |
| *N.samayunkur* | 1,401 | 43.11 | 9.85 | 37.76 | 9.28 | 19.13 | 80.87 | 0.07 | 0.03 |
| *H.aculeatus* | 1,462 | 44.46 | 8.48 | 40.56 | 6.50 | 14.98 | 85.02 | 0.05 | 0.13 |
| *T. palmi* | 1,393 | 42.21 | 10.12 | 38.05 | 9.62 | 19.74 | 80.26 | 0.05 | 0.03 |
| *T. imaginis* | 1,492 | 43.83 | 9.45 | 36.66 | 10.05 | 19.50 | 80.50 | 0.09 | -0.03 |
| *F. intonsa* | 1,392 | 43.53 | 10.70 | 35.78 | 9.99 | 20.69 | 79.31 | 0.10 | 0.03 |
| *F. occidentalis* | 1,380 | 42.39 | 10.58 | 37.39 | 9.64 | 20.22 | 79.78 | 0.06 | 0.05 |
| *S. dorsalis* EA1 | 1,426 | 40.53 | 11.01 | 37.52 | 10.94 | 21.95 | 78.05 | 0.04 | 0.00 |
| *S. dorsalis* SA1 | 1,429 | 41.36 | 10.43 | 38.21 | 10.01 | 20.43 | 79.57 | 0.04 | 0.02 |
| *A. obscurus* | 1,430 | 39.79 | 10.63 | 39.86 | 9.72 | 20.35 | 79.65 | 0.00 | 0.04 |
| **Ribosomal RNA** | | | | | | | | | |
| *F.vespiformis* | 1,071 | 41.83 | 14.19 | 33.05 | 10.92 | 25.12 | 74.88 | 0.12 | 0.13 |
| *G.uzeli* | 1,384 | 43.21 | 9.90 | 39.60 | 7.30 | 17.20 | 82.80 | 0.04 | 0.15 |
| *R.cruentatus* | 1,796 | 44.60 | 9.52 | 34.63 | 11.25 | 20.77 | 79.23 | 0.13 | -0.08 |
| *H.indicus* | 1,759 | 43.21 | 11.54 | 34.00 | 11.26 | 22.80 | 77.20 | 0.12 | 0.01 |
| *D.minowai* | 1,842 | 45.49 | 11.18 | 34.09 | 9.23 | 20.41 | 79.59 | 0.14 | 0.10 |
| *N.samayunkur* | 1,808 | 44.97 | 11.73 | 34.35 | 8.96 | 20.69 | 79.31 | 0.13 | 0.13 |
| *H.aculeatus* | 1,882 | 42.51 | 8.50 | 42.67 | 6.32 | 14.82 | 85.18 | 0.00 | 0.15 |
| *T. palmi* | 1,870 | 47.38 | 10.7 | 32.83 | 9.09 | 19.79 | 80.21 | 0.18 | 0.08 |
| *T. imaginis* | 1,876 | 47.65 | 10.77 | 32.14 | 9.43 | 20.2 | 79.8 | 0.19 | 0.07 |
| *F. intonsa* | 1,699 | 47.15 | 11.3 | 32.02 | 9.54 | 20.84 | 79.16 | 0.19 | 0.08 |
| *F. occidentalis* | 1,848 | 45.94 | 12.18 | 33.93 | 7.95 | 20.13 | 79.87 | 0.15 | 0.21 |
| *S. dorsalis* EA1 | 1,775 | 43.21 | 11.89 | 34.99 | 9.92 | 21.8 | 78.2 | 0.11 | 0.09 |
| *S. dorsalis* SA1 | 1,777 | 45.36 | 11.65 | 34.44 | 8.55 | 20.2 | 79.8 | 0.14 | 0.15 |
| *A. obscurus* | 1,812 | 43.16 | 11.7 | 36.59 | 8.55 | 20.25 | 79.75 | 0.08 | 0.16 |
| **Control Region** | | | | | | | | | |
| *F.vespiformis* | 1,841 | 34.98 | 12.11 | 40.25 | 12.66 | 24.77 | 75.23 | -0.07 | -0.02 |
| *G.uzeli* | 502 | 45.82 | 5.18 | 44.62 | 4.38 | 9.56 | 90.44 | 0.01 | 0.08 |
| *R.cruentatus* | 985 | 42.13 | 10.25 | 32.59 | 15.03 | 25.28 | 74.72 | 0.13 | -0.19 |
| *H.indicus* | 493 | 34.48 | 11.16 | 40.97 | 13.39 | 24.54 | 75.46 | -0.09 | -0.09 |
| *D.minowai* | 149 | 38.26 | 3.36 | 53.69 | 4.70 | 8.05 | 91.95 | -0.17 | -0.17 |
| *N.samayunkur* | 928 | 33.84 | 14.87 | 37.28 | 14.01 | 28.88 | 71.12 | -0.05 | 0.03 |
| *H.aculeatus* | 493 | 34.48 | 11.16 | 40.97 | 13.39 | 24.54 | 75.46 | -0.09 | -0.09 |
| *T. palmi* | 627 | 44.82 | 4.63 | 40.99 | 9.57 | 14.19 | 85.81 | 0.04 | -0.35 |
| *T. imaginis* | 900 | 47.56 | 16.67 | 25.22 | 10.56 | 27.22 | 72.78 | 0.31 | 0.22 |
| *F. intonsa* | 942 | 41.72 | 7.86 | 38.22 | 12.21 | 20.06 | 79.94 | 0.04 | -0.22 |
| *F. occidentalis* | 595 | 40.34 | 7.90 | 43.70 | 8.07 | 15.97 | 84.03 | -0.04 | -0.01 |
| *S. dorsalis* EA1 | 1,775 | 43.21 | 11.89 | 34.99 | 9.92 | 21.80 | 78.20 | 0.11 | 0.09 |
| *S. dorsalis* SA1 | 767 | 35.33 | 9.26 | 43.55 | 11.86 | 21.12 | 78.88 | -0.10 | -0.12 |
| *A. obscurus* | 145 | 25.52 | 8.97 | 62.76 | 2.76 | 11.72 | 88.28 | -0.42 | 0.53 |

**Table S8. Pairwise-genetic difference of orthologous *tRNAs* in thrips mitogenomes.**

| **Sl.NO.** | **tRNA** | **BDPS** | **PDIST** | **MLDIST** |
| --- | --- | --- | --- | --- |
|  | *trnA* | 13.54 | 0.251±0.09 | 0.385±0.19 |
|  | *trnC* | 14.24 | 0.303±0.12 | 0.516±0.31 |
|  | *trnD* | 19.04 | 0.359±0.09 | 0.592±0.24 |
|  | *trnE* | 6.363 | 0.163±0.07 | 0.293±0.18 |
|  | *trnF* | 10.15 | 0.195±0.07 | 0.267±0.12 |
|  | *trnG* | 14.40 | 0.294±0.09 | 0.472±0.21 |
|  | *trnH* | 10.67 | 0.209±0.07 | 0.291±0.13 |
|  | *trnI* | 4.31 | 0.251±0.08 | 0.332±0.13 |
|  | *trnK* | 13.25 | 0.288±0.10 | 0.435±0.20 |
|  | *trnL1* | 14.22 | 0.279±0.12 | 0.439±0.26 |
|  | *trnL2* | 18.967 | 0.365±0.16 | 0.861±0.74 |
|  | *trnM* | 9.90 | 0.202±0.10 | 0.261±0.16 |
|  | *trnN* | 10.77 | 0.250±0.09 | 0.358±0.19 |
|  | *trnP* | 13.33 | 0.252±0.08 | 0.362±0.15 |
|  | *trnQ* | 12.21 | 0.244±0.13 | 0.413±0.29 |
|  | *trnR* | 14.30 | 0.280±0.11 | 0.388±0.20 |
|  | *trnS1* | 15.32 | 0.383±0.08 | 0.606±0.20 |
|  | *trnS2* | 8.25 | 0.153± 0.09 | 0.198±0.14 |
|  | *trnT* | 17.20 | 0.313±0.08 | 0.521±0.17 |
|  | *trnV* | 14.17 | 0.373±0.12 | 0.744±0.36 |
|  | *trnW* | 14.74 | 0.254±0.12 | 0.389±0.23 |
|  | *trnY* | 14.74 | 0.254±0.12 | 0.389±0.23 |

**Table S9. The output file of TreeREx result of thrips species mitogenome using protein coding genes, ribosomal RNA and control region one.**

# testing putative ancestral states

# cox1 cox2 atp8 atp6 CR rrnL nad2 rrnS nad4L nad4 -cytb nad5 -nad3 -cox3 -nad6 nad1

# cox1 cox2 cox3 nad3 cytb nad2 nad1 rrnS atp8 atp6 CR -nad5 -nad4 -nad4L nad6 rrnL

# score 4

# testing putative ancestral states

# cox1 cox2 atp8 atp6 CR rrnL nad2 rrnS nad4L nad4 -cytb nad5 -nad3 -cox3 -nad6 nad1

# cox1 cytb nad2 nad1 atp8 atp6 cox2 cox3 nad3 rrnS CR -nad5 -nad4 -nad4L nad6 rrnL

# score 5

# testing putative ancestral states

# cox1 cox2 atp8 atp6 CR rrnL nad2 rrnS nad4L nad4 -cytb nad5 -nad3 -cox3 -nad6 nad1

# cox1 nad1 cox2 cox3 nad3 rrnS CR cytb nad2 atp8 atp6 -nad5 -nad4 -nad4L nad6 rrnL

# score 5

# testing putative ancestral states

# cox1 cox2 atp8 atp6 CR rrnL nad2 rrnS nad4L nad4 -cytb nad5 -nad3 -cox3 -nad6 nad1

# cox1 cox2 cox3 nad3 cytb nad2 nad1 rrnS atp8 atp6 CR -nad5 -nad4 -nad4L nad6 rrnL

# score 4

# testing putative ancestral states

# cox1 cox2 atp8 atp6 CR rrnL nad2 rrnS nad4L nad4 -cytb nad5 -nad3 -cox3 -nad6 nad1

# cox1 cytb nad2 nad1 atp8 atp6 cox2 cox3 nad3 rrnS CR -nad5 -nad4 -nad4L nad6 rrnL

# score 5

# testing putative ancestral states

# cox1 cox2 atp8 atp6 CR rrnL nad2 rrnS nad4L nad4 -cytb nad5 -nad3 -cox3 -nad6 nad1

# cox1 nad1 cox2 cox3 nad3 rrnS CR cytb nad2 atp8 atp6 -nad5 -nad4 -nad4L nad6 rrnL

# score 5

# testing putative ancestral states

# cox1 cox2 atp8 atp6 CR rrnL nad2 rrnS nad4L nad4 -cytb nad5 -nad3 -cox3 -nad6 nad1

# cox1 nad1 cox2 cox3 nad3 rrnS CR cytb nad2 atp8 atp6 -nad5 -nad4 -nad4L nad6 rrnL

# score 5

# testing putative ancestral states

# cox1 cox2 atp8 atp6 CR rrnL nad2 rrnS nad4L nad4 -cytb nad5 -nad3 -cox3 -nad6 nad1

# cox1 cox2 cox3 nad3 CR cytb nad2 nad1 rrnS atp8 atp6 -nad5 -nad4 -nad4L nad6 rrnL

# score 4

# testing putative ancestral states

# cox1 cox2 atp8 atp6 CR rrnL nad2 rrnS nad4L nad4 -cytb nad5 -nad3 -cox3 -nad6 nad1

# cox1 cox2 cox3 nad3 cytb nad2 nad1 rrnS atp8 atp6 CR -nad5 -nad4 -nad4L nad6 rrnL

# score 4

# testing putative ancestral states

# cox1 cox2 atp8 atp6 CR rrnL nad2 rrnS nad4L nad4 -cytb nad5 -nad3 -cox3 -nad6 nad1

# cox1 nad1 cox2 cox3 nad3 rrnS CR cytb nad2 atp8 atp6 -nad5 -nad4 -nad4L nad6 rrnL

# score 5

# testing putative ancestral states

# cox1 cox2 atp8 atp6 CR rrnL nad2 rrnS nad4L nad4 -cytb nad5 -nad3 -cox3 -nad6 nad1

# cox1 cox2 cox3 nad3 CR cytb nad2 nad1 rrnS atp8 atp6 -nad5 -nad4 -nad4L nad6 rrnL

# score 4

# testing putative ancestral states

# cox1 cox2 atp8 atp6 CR rrnL nad2 rrnS nad4L nad4 -cytb nad5 -nad3 -cox3 -nad6 nad1

# cox1 cox2 cox3 nad3 cytb nad2 nad1 rrnS atp8 atp6 CR -nad5 -nad4 -nad4L nad6 rrnL

# score 4

minscore=4 2nd minscore 4

minscore=4 2nd minscore 4

minscore=4 2nd minscore 4

# testing putative ancestral states

# cox1 cox2 atp8 atp6 CR rrnL nad2 rrnS nad4L nad4 -cytb nad5 -nad3 -cox3 -nad6 nad1

# cox1 cox2 atp8 atp6 cox3 nad3 -nad5 -nad4 -nad4L nad6 cytb -nad1 -rrnL -rrnS CR nad2

# score 7

# testing putative ancestral states

# cox1 cox2 atp8 atp6 CR rrnL nad2 rrnS nad4L nad4 -cytb nad5 -nad3 -cox3 -nad6 nad1

# cox1 cox2 atp8 atp6 cox3 nad3 -nad5 -nad4 -nad4L nad6 cytb -nad1 -rrnL -rrnS CR nad2

# score 7

# testing putative ancestral states

# cox1 cox2 atp8 atp6 CR rrnL nad2 rrnS nad6 cox3 nad3 -nad5 cytb -nad4 -nad4L nad1

# cox1 cox2 atp8 atp6 cox3 nad3 -nad5 -nad4 -nad4L nad6 cytb -nad1 -rrnL -rrnS CR nad2

# score 6

# testing putative ancestral states

# cox1 cox2 atp8 atp6 CR rrnL nad2 rrnS nad6 cox3 nad3 -nad5 cytb -nad4 -nad4L nad1

# cox1 cox2 atp8 atp6 cox3 nad3 -nad5 -nad4 -nad4L nad6 cytb -nad1 -rrnL -rrnS CR nad2

# score 6

# testing putative ancestral states

# cox1 cox2 rrnS nad6 cox3 nad3 cytb atp8 atp6 CR rrnL nad2 -nad5 -nad4 -nad4L nad1

# cox1 cox2 atp8 atp6 cox3 nad3 -nad5 -nad4 -nad4L nad6 cytb -nad1 -rrnL -rrnS CR nad2

# score 4

# testing putative ancestral states

# cox1 cox2 rrnS nad6 cox3 nad3 cytb atp8 atp6 CR rrnL nad2 -nad5 -nad4 -nad4L nad1

# cox1 cox2 atp8 atp6 cox3 nad3 -nad5 -nad4 -nad4L nad6 cytb -nad1 -rrnL -rrnS CR nad2

# score 4

# testing putative ancestral states

# cox1 cox2 nad6 cox3 nad3 cytb rrnL nad2 nad1 rrnS atp8 atp6 CR -nad5 -nad4 -nad4L

# cox1 cox2 atp8 atp6 cox3 nad3 -nad5 -nad4 -nad4L nad6 cytb -nad1 -rrnL -rrnS CR nad2

# score 4

# testing putative ancestral states

# cox1 cox2 nad6 cox3 nad3 cytb rrnL nad2 nad1 rrnS atp8 atp6 CR -nad5 -nad4 -nad4L

# cox1 cox2 atp8 atp6 cox3 nad3 -nad5 -nad4 -nad4L nad6 cytb -nad1 -rrnL -rrnS CR nad2

# score 4

# testing putative ancestral states

# cox1 cox2 cox3 nad3 cytb nad2 nad1 rrnS atp8 atp6 CR -nad5 -nad4 -nad4L nad6 rrnL

# cox1 cox2 atp8 atp6 cox3 nad3 -nad5 -nad4 -nad4L nad6 cytb -nad1 -rrnL -rrnS CR nad2

# score 4

# testing putative ancestral states

# cox1 cox2 cox3 nad3 cytb nad2 nad1 rrnS atp8 atp6 CR -nad5 -nad4 -nad4L nad6 rrnL

# cox1 cox2 atp8 atp6 cox3 nad3 -nad5 -nad4 -nad4L nad6 cytb -nad1 -rrnL -rrnS CR nad2

# score 4

minscore=4 2nd minscore 4

# testing putative ancestral states

# cox1 cox2 cox3 nad3 cytb nad2 nad1 rrnS atp8 atp6 CR -nad5 -nad4 -nad4L nad6 rrnL

# cox1 cox2 atp8 atp6 cox3 nad3 -nad5 -nad4 -nad4L nad6 cytb -nad1 -rrnL -rrnS CR nad2

# score 4

# testing putative ancestral states

# cox1 cox2 cox3 nad3 cytb nad2 nad1 rrnS atp8 atp6 CR -nad5 -nad4 -nad4L nad6 rrnL

# cox1 cox2 atp8 atp6 cox3 nad3 -nad5 -nad4 -nad4L nad6 cytb -nad1 -rrnL -rrnS CR nad2

# score 4

# testing putative ancestral states

# cox1 cox2 cox3 nad3 cytb nad2 nad1 rrnS atp8 atp6 CR -nad5 -nad4 -nad4L nad6 rrnL

# cox1 cox2 atp8 atp6 cox3 nad3 -nad5 -nad4 -nad4L nad6 cytb -nad1 -rrnL -rrnS CR nad2

# score 4

# testing putative ancestral states

# cox1 cox2 cox3 nad3 cytb nad2 nad1 rrnS atp8 atp6 CR -nad5 -nad4 -nad4L nad6 rrnL

# cox1 cox2 atp8 atp6 cox3 nad3 -nad5 -nad4 -nad4L nad6 cytb -nad1 -rrnL -rrnS CR nad2

# score 4

minscore=4 2nd minscore 4

minscore=4 2nd minscore 4

>A13

>A12

cox1 cox2 rrnS nad6 cox3 nad3 cytb atp8 atp6 CR rrnL nad2 -nad5 -nad4 -nad4L nad1

>A0

cox1 cox2 atp8 atp6 CR rrnL nad2 rrnS nad4L nad4 -cytb nad5 -nad3 -cox3 -nad6 nad1

>GU

cox1 cox2 atp8 atp6 cox3 nad3 -nad5 -nad4 -nad4L nad6 cytb -rrnS -rrnL CR -nad2 nad1

>HA

cox1 cox2 atp8 atp6 CR rrnL nad2 rrnS nad4L nad4 -cytb nad5 -nad3 -cox3 -nad6 nad1

>A11

cox1 cox2 cox3 nad3 cytb nad2 nad1 rrnS atp8 atp6 CR -nad5 -nad4 -nad4L nad6 rrnL

>A10

cox1 cox2 cox3 nad3 cytb nad2 nad1 rrnS atp8 atp6 CR -nad5 -nad4 -nad4L nad6 rrnL

>A9

cox1 cox2 cox3 nad3 cytb nad2 nad1 rrnS atp8 atp6 CR -nad5 -nad4 -nad4L nad6 rrnL

>A8

cox1 cox2 cox3 nad3 cytb nad2 nad1 rrnS atp8 atp6 CR -nad5 -nad4 -nad4L nad6 rrnL

>A1

cox1 cox2 cox3 nad3 cytb nad2 nad1 rrnS atp8 atp6 CR -nad5 -nad4 -nad4L nad6 rrnL

>AO

cox1 cox2 cox3 nad3 cytb nad2 nad1 rrnS atp8 atp6 CR -nad5 -nad4 -nad4L nad6 rrnL

>DM

cox1 cox2 cox3 nad3 cytb nad2 nad1 rrnS atp8 atp6 CR -nad5 -nad4 -nad4L nad6 rrnL

>A7

cox1 nad3 cox2 cox3 cytb nad2 nad1 rrnS atp8 atp6 CR -nad5 -nad4 -nad4L nad6 rrnL

>A4

cox1 nad3 cox2 cox3 cytb nad2 nad1 rrnS atp8 atp6 CR -nad5 -nad4 -nad4L nad6 rrnL

>A2

cox1 nad3 cox2 cox3 cytb nad2 nad1 rrnS atp8 atp6 CR -nad5 -nad4 -nad4L nad6 rrnL

>FI

cox1 nad3 cox2 cox3 cytb nad2 nad1 rrnS atp8 atp6 CR -nad5 -nad4 -nad4L nad6 rrnL

>FO

cox1 nad3 cox2 cox3 cytb nad2 nad1 rrnS atp8 atp6 CR -nad5 -nad4 -nad4L nad6 rrnL

>A3

cox1 nad3 cox2 cox3 cytb nad2 nad1 rrnS atp8 atp6 CR -nad5 -nad4 -nad4L nad6 rrnL

>TI

cox1 nad3 cox2 cox3 cytb nad2 nad1 rrnS atp8 atp6 CR -nad5 -nad4 -nad4L nad6 rrnL

>TP

cox1 nad3 cox2 cox3 cytb nad2 nad1 rrnS atp8 atp6 CR -nad5 -nad4 -nad4L nad6 rrnL

>A6

cox1 nad3 cox2 cox3 cytb nad2 nad1 rrnS atp8 atp6 CR -nad5 -nad4 -nad4L nad6 rrnL

>NS

cox1 nad3 cox2 cox3 cytb nad2 nad1 rrnS -atp8 atp6 -nad5 -nad4 -nad4L CR nad6 rrnL

>A5

cox1 nad3 cox2 cox3 cytb nad2 nad1 rrnS atp8 atp6 CR -nad5 -nad4 -nad4L nad6 rrnL

>SDE

cox1 nad3 cox2 cox3 cytb nad2 nad1 rrnS atp8 atp6 CR -nad5 -nad4 -nad4L nad6 rrnL

>SDS

cox1 nad3 cox2 cox3 cytb nad2 nad1 rrnS atp8 atp6 CR -nad5 -nad4 -nad4L rrnL nad6

>RC

cox1 cox2 cox3 nad3 cytb nad2 nad1 rrnS atp8 atp6 CR -nad5 -nad4 -nad4L nad6 rrnL

>HI

cox1 cox2 cox3 nad3 cytb nad2 nad1 rrnS atp8 atp6 CR -nad5 -nad4 -nad4L nad6 rrnL

>FV

cox1 nad1 cox2 cox3 nad3 rrnS CR cytb nad2 atp8 atp6 -nad5 -nad4 -nad4L nad6 rrnL

>AB

cox1 cox2 atp8 atp6 cox3 nad3 -nad5 -nad4 -nad4L nad6 cytb -nad1 -rrnL -rrnS CR nad2

scenario to A13

Empty

scenario to A12

Empty

scenario to A0

alternative(

ordered[

inversion(atp8 atp6 cox3 nad3 nad5 nad4 nad4L nad6 cytb rrnL CR nad2 )

, inversion(atp8 atp6 rrnL CR nad2 )

, TDRL(atp8 atp6 cox3 nad3 nad5 nad6 rrnL CR nad2 ,nad4 nad4L cytb rrnS ,)

, TDRL(atp8 atp6 nad4 nad4L cytb rrnL rrnS CR nad2 ,cox3 nad3 nad5 nad6 ,)

] complete=1

, ordered[

inversion(cox3 nad3 nad6 cytb )

, inversion(nad5 nad4 nad4L )

, TDRL(atp8 atp6 nad5 cytb rrnL CR nad2 ,cox3 nad3 nad4 nad4L nad6 rrnS ,)

, TDRL(atp8 atp6 nad4 nad4L rrnL rrnS CR nad2 ,cox3 nad3 nad5 nad6 cytb ,)

] complete=1

,) complete=1

scenario to GU

unordered{

TDRL(nad6 cytb ,cox3 nad3 nad5 nad4 nad4L ,)

, inversion(nad2 )

, inverse transposition(cox3 nad3 nad5 nad4 nad4L nad6 cytb rrnS ,rrnL CR nad2 ,)

, inverse transposition(rrnL ,CR ,)

,} complete=1

scenario to HA

Empty

scenario to A11

ordered[

TDRL(cox3 nad3 nad6 cytb nad1 rrnL nad2 ,atp8 atp6 nad5 nad4 nad4L rrnS CR ,)

, TDRL(atp8 atp6 cox3 nad3 nad5 nad4 nad4L cytb nad1 rrnS CR nad2 ,nad6 rrnL ,)

] complete=1

scenario to A10

Empty

scenario to A9

Empty

scenario to A8

Empty

scenario to A1

Empty

scenario to AO

Empty

scenario to DM

Empty

scenario to A7

transposition(cox2 cox3 ,nad3 ,)

scenario to A4

Empty

scenario to A2

Empty

scenario to FI

Empty

scenario to FO

Empty

scenario to A3

Empty

scenario to TI

Empty

scenario to TP

Empty

scenario to A6

Empty

scenario to NS

unordered{

transposition(nad5 nad4 nad4L ,CR ,)

, inversion(atp8 )

,} complete=1

scenario to A5

Empty

scenario to SDE

Empty

scenario to SDS

transposition(nad6 ,rrnL ,)

scenario to RC

Empty

scenario to HI

Empty

scenario to FV

ordered[

TDRL(atp8 atp6 cytb nad1 nad2 ,cox2 cox3 nad3 rrnS ,)

, TDRL(cox2 cox3 nad3 nad1 rrnS CR ,atp8 atp6 cytb nad2 ,)

] complete=1

scenario to AB

Empty

**Figures S1**. **Comparative secondary structure of tRNA families in thrips mitochondrial genomes**. The nucleotide substitution pattern for each tRNA family was modelled using the *A*. *obscurus* tRNA as the structural reference. (A) Alanine, Arginine, Aspartic Acid, Asparagine, Cysteine, Glycine, Glutamic Acid, Glutamine, Histidine, Isoleucine, Leucine 1 and 2.


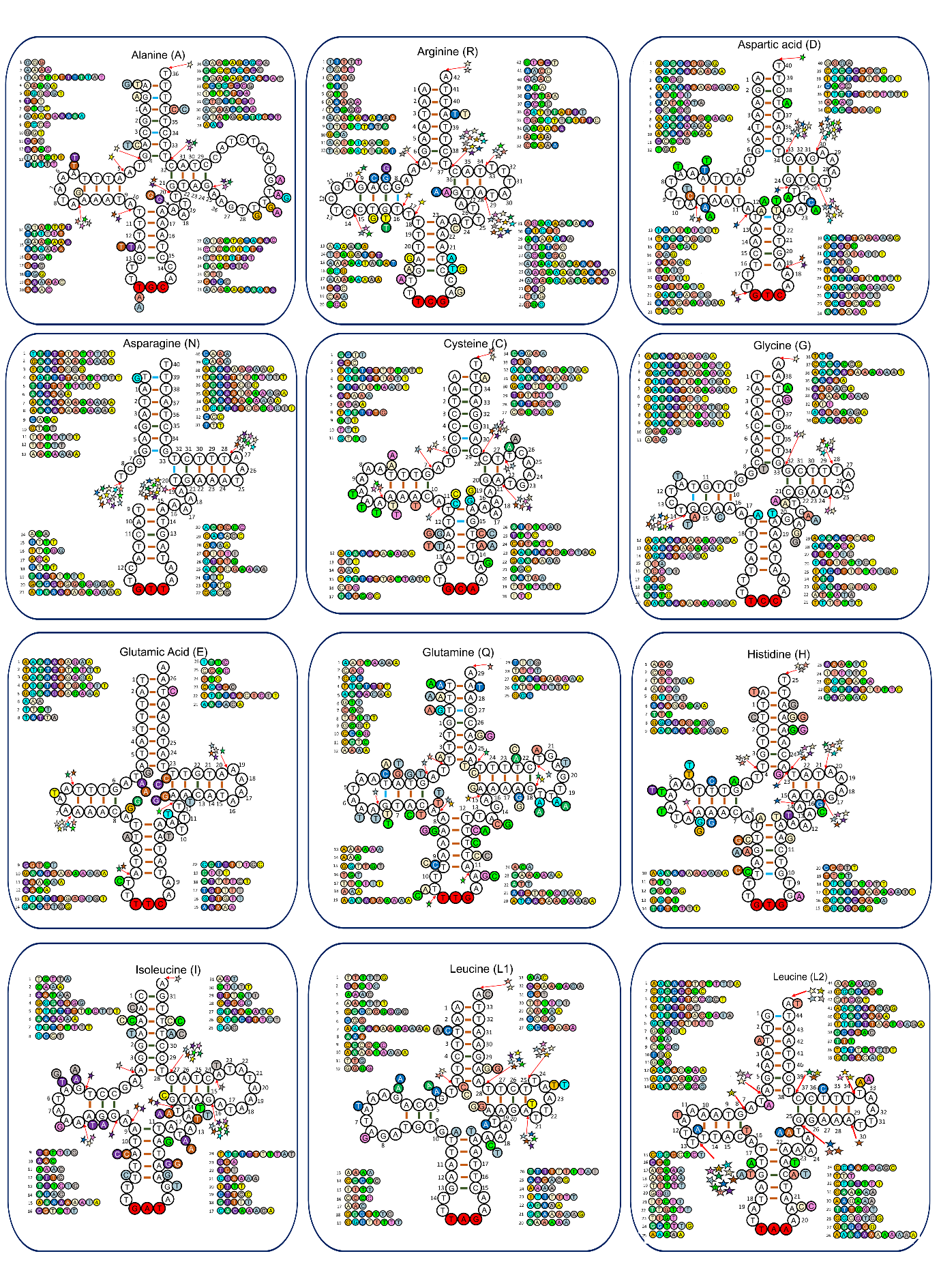


**Figures S2**. **Comparative secondary structure of tRNA families in thrips mitochondrial genomes**. The nucleotide substitution pattern for each tRNA family was modelled using the *A*. *obscurus* tRNA as the structural reference. (B) Lysine, Methionine, Phenylalanine, Proline, Serine 1 and 2, Threonine, Tyrosine, Tryptophan, Valine with
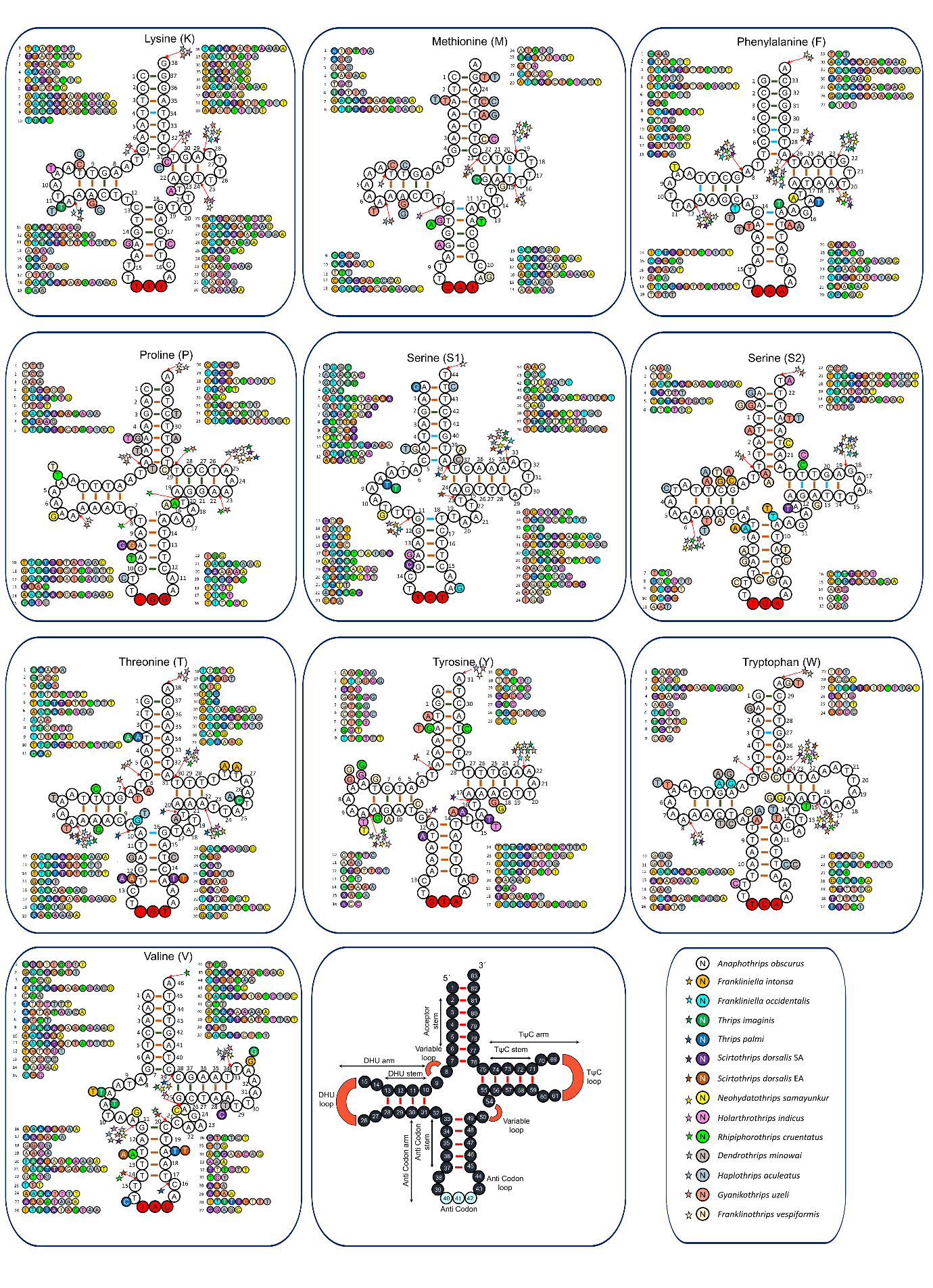
hypothetical tRNA with structural details.

**Fig. S3.** **Bayesian Phylogenetic tree (BI-2) inferred by PCGs**. The Bayesian posterior probabilities (BI-2) and bootstrap support (ML-1) are superimposed on each node. The tree is drawn to scale with values indicated along with the branches.

**
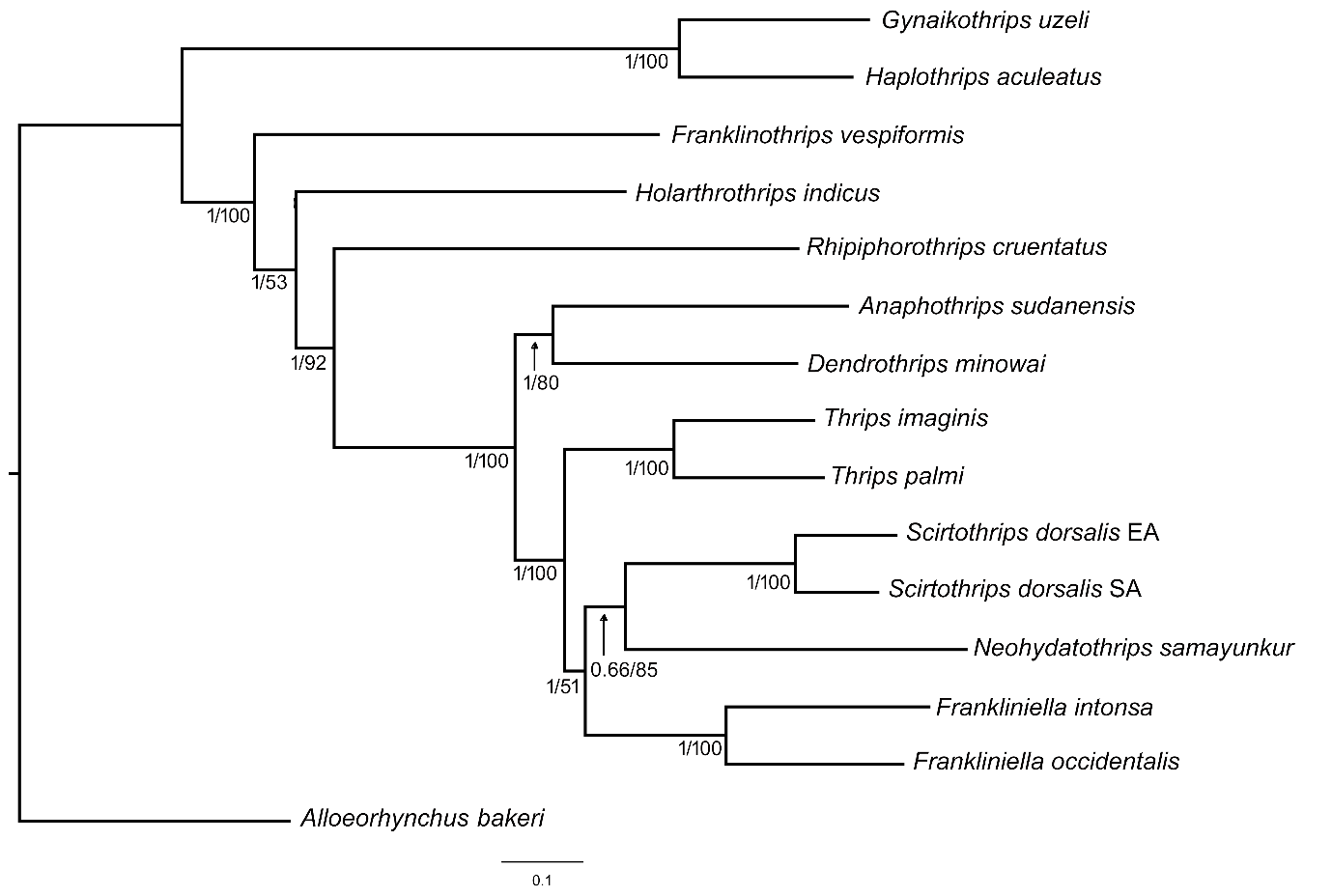
**

**Fig.** **S4**. **Bayesian Phylogenetic tree (BI-3) inferred by PCGs**. The Bayesian posterior probabilities (BI-3) and bootstrap support (ML-3) are superimposed on each node. The tree is drawn to scale with values indicated along with the branches.


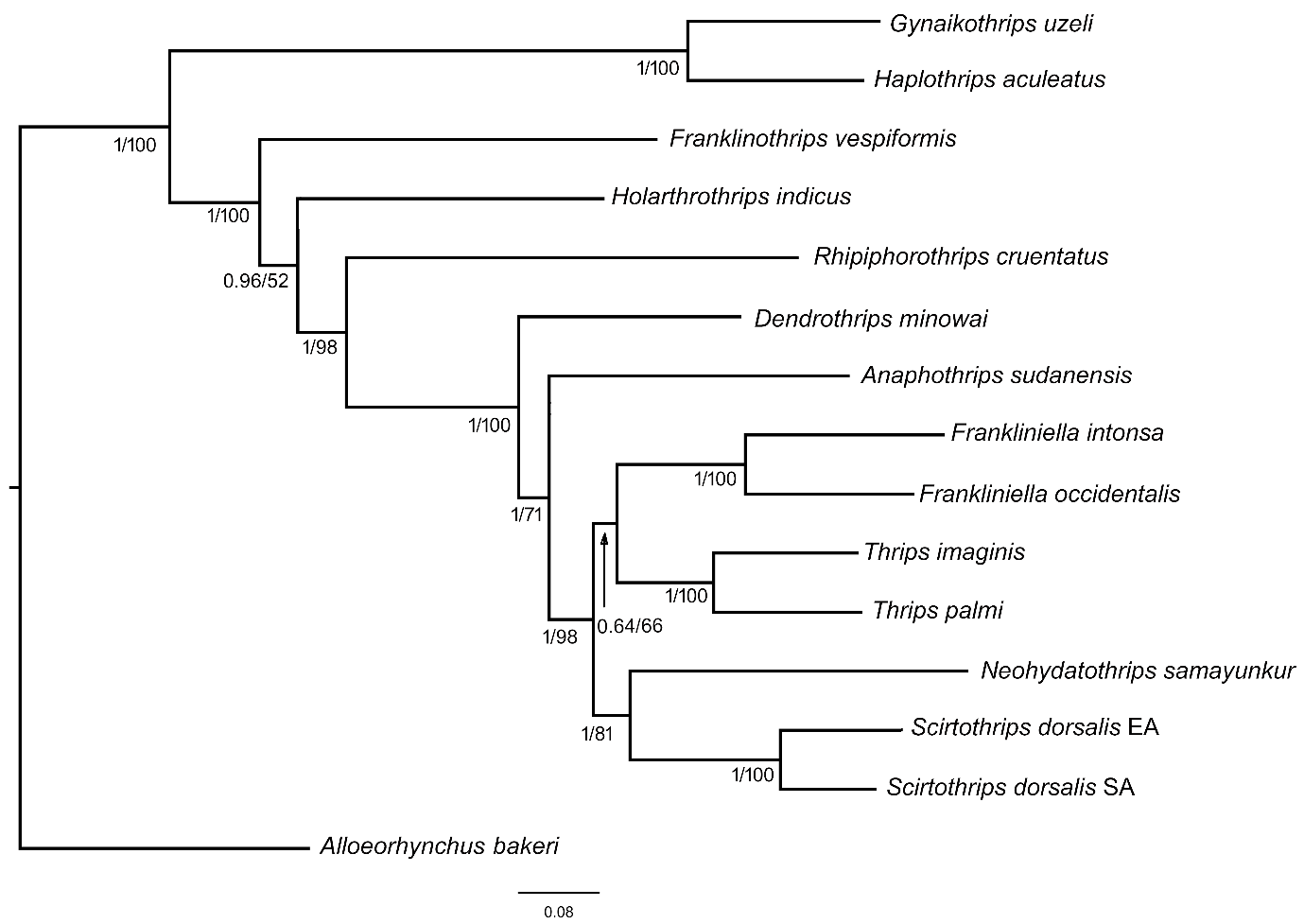


**Fig. S5.** **Maximum likelihood (ML-2) inferred by PCGs**. The tree is drawn to scale with values indicated along with the branches.


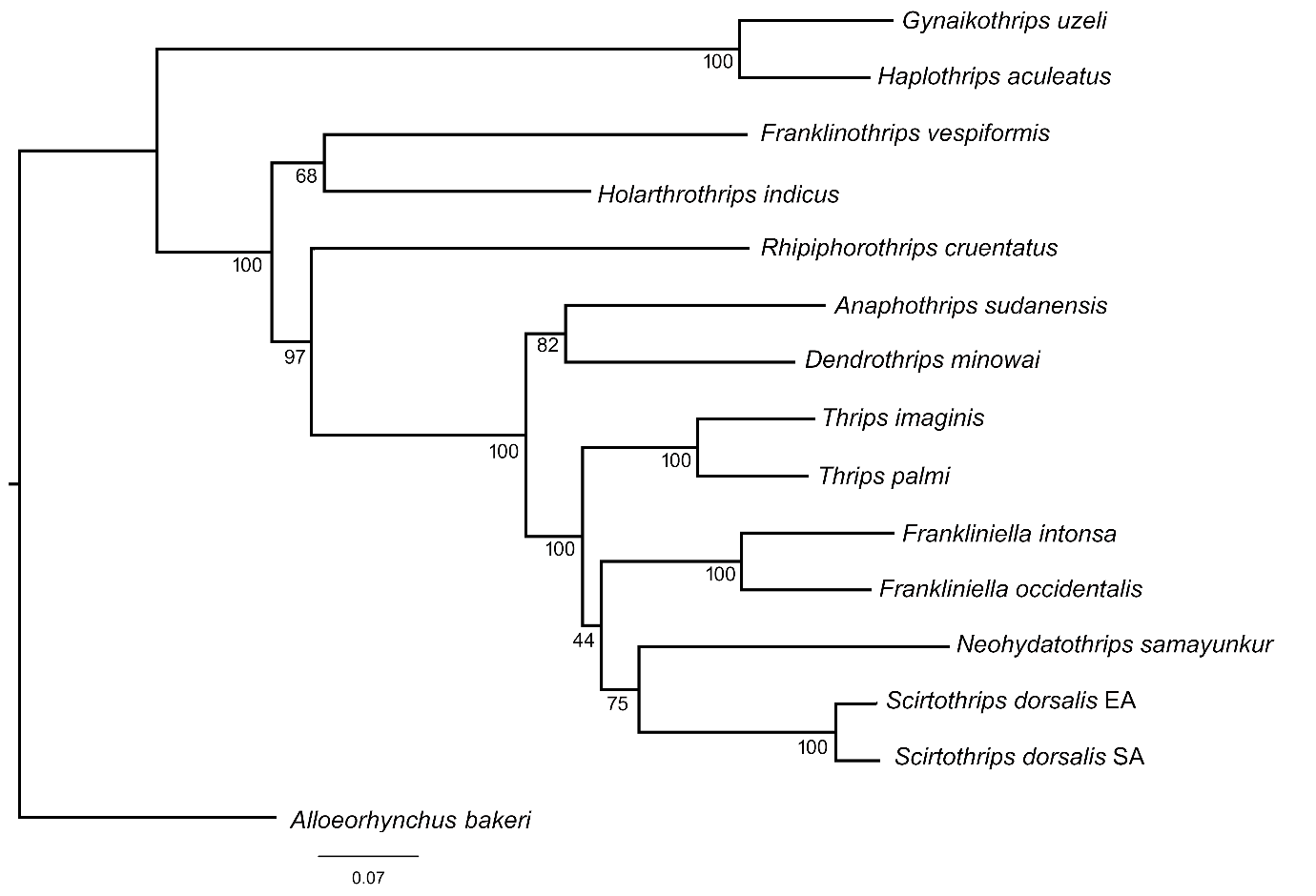


**Fig. S6.** **Maximum likelihood (ML-4) inferred by PCGs**. The tree is drawn to scale with values indicated along with the branches.


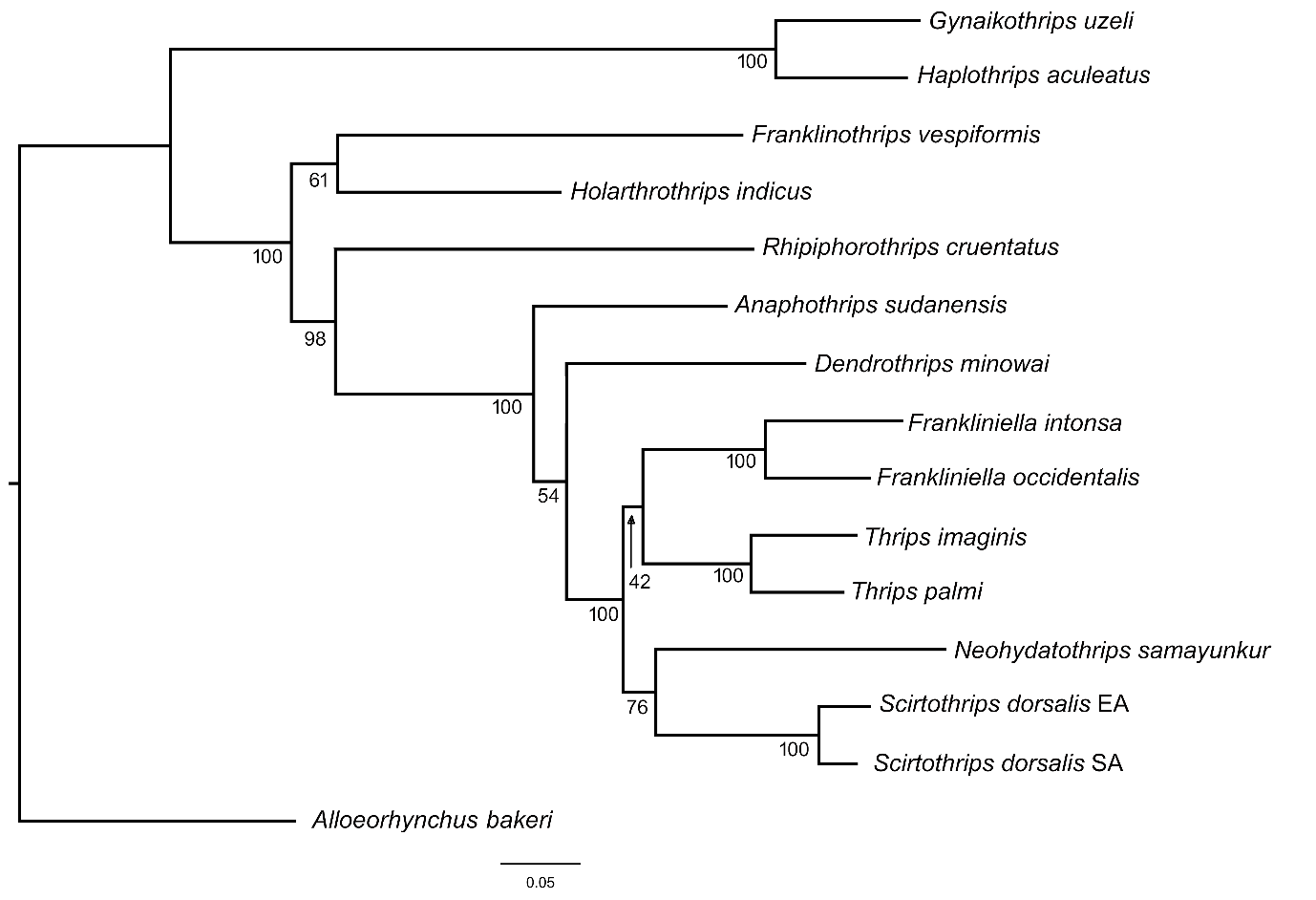


F**igure S7. Evolution of gene order in mitochondrial genome explained by CREx**. Rearrangement operations occurred from inferred ancestral insect gene order to *F*. *vespiformis*, *H*. *indicus, R*. *cruentatus*.


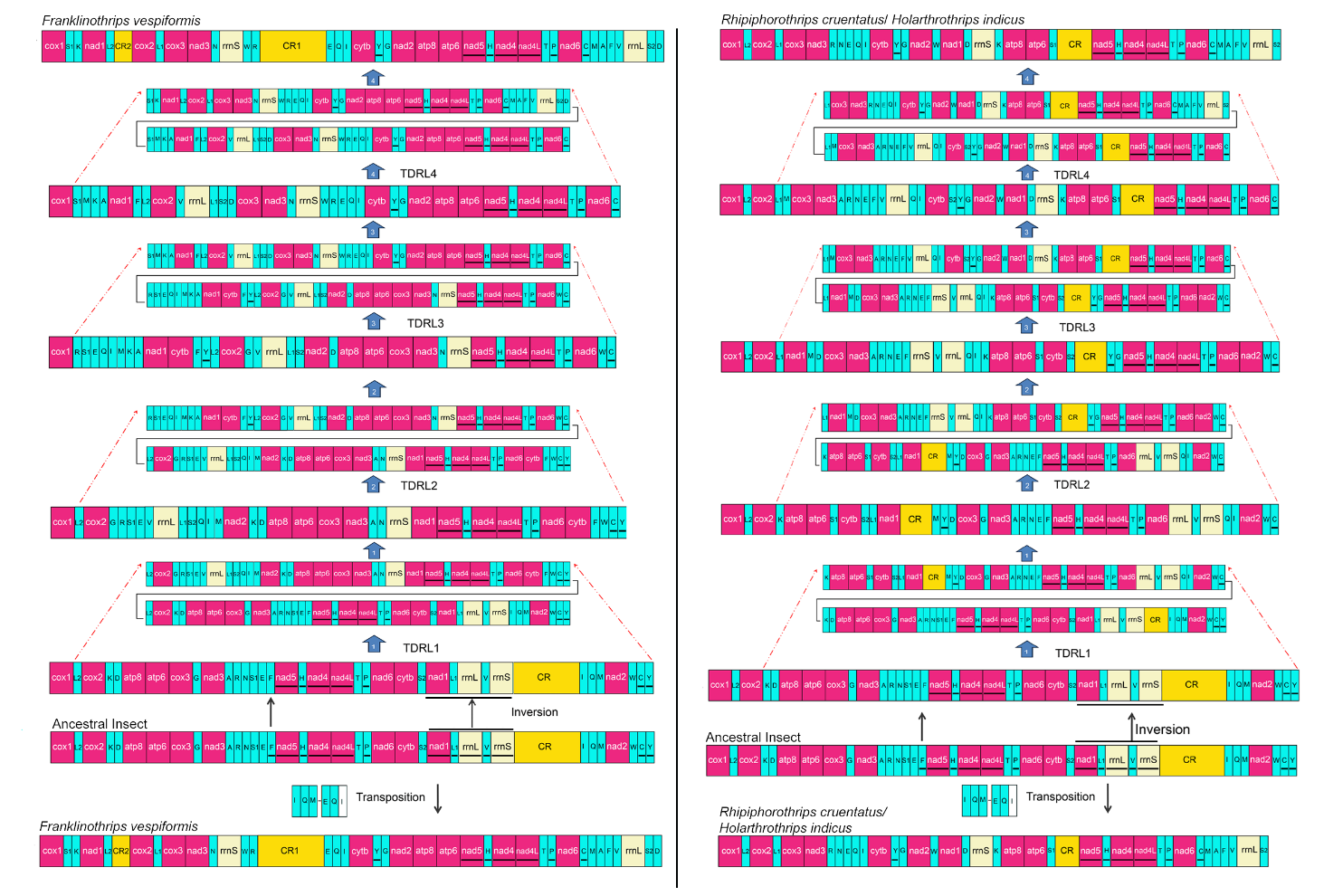


F**igure S8. Evolution of gene order in mitochondrial genome explained by CREx**. Rearrangement operations occurred from inferred ancestral insect gene order to *G*. *uzeli*.


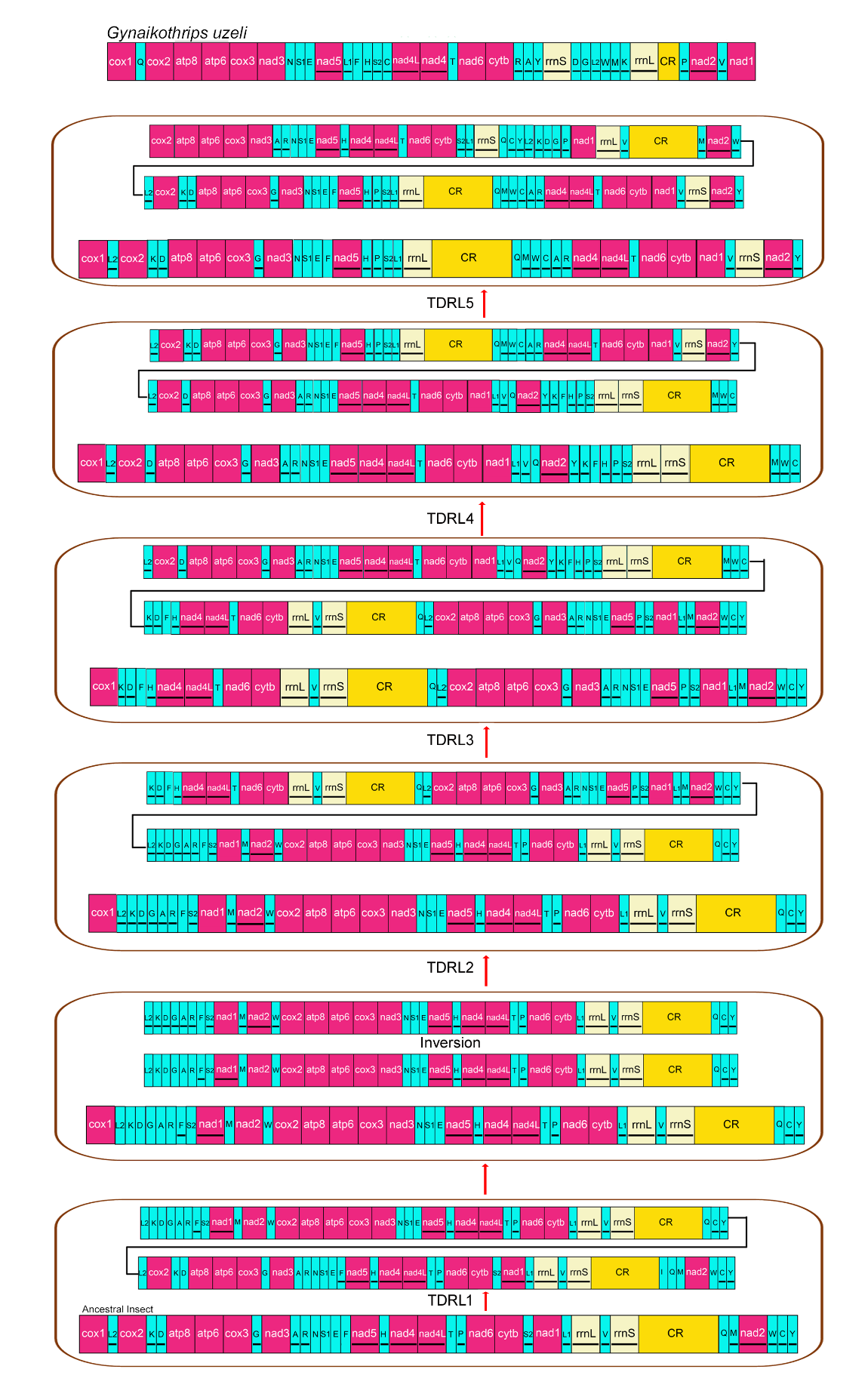

Supplement: Supplementary file 1 — Supplementary Info. [file 41598_2020_57705_MOESM1_ESM.docx]
